# Identifying the nature of the active sites in methanol synthesis over Cu/ZnO/Al_2_O_3_ catalysts

**DOI:** 10.1038/s41467-020-17631-5

**Published:** 2020-08-04

**Authors:** Daniel Laudenschleger, Holger Ruland, Martin Muhler

**Affiliations:** 1 Laboratory of Industrial Chemistry, Ruhr University Bochum, Universitätsstraße 150, D-44780 Bochum, Germany; 2 Max Planck Institute for Chemical Energy Conversion, Stiftstraße 34-36, D-45470 Mülheim an der Ruhr, Germany

**Keywords:** Catalytic mechanisms, Heterogeneous catalysis, Chemical hydrogen storage

## Abstract

The heterogeneously catalysed reaction of hydrogen with carbon monoxide and carbon dioxide (syngas) to methanol is nearly 100 years old, and the standard methanol catalyst Cu/ZnO/Al_2_O_3_ has been applied for more than 50 years. Still, the nature of the Zn species on the metallic Cu^0^ particles (interface sites) is heavily debated. Here, we show that these Zn species are not metallic, but have a positively charged nature under industrial methanol synthesis conditions. Our kinetic results are based on a self-built high-pressure pulse unit, which allows us to inject selective reversible poisons into the syngas feed passing through a fixed-bed reactor containing an industrial Cu/ZnO/Al_2_O_3_ catalyst under high-pressure conditions. This method allows us to perform surface-sensitive operando investigations as a function of the reaction conditions, demonstrating that the rate of methanol formation is only decreased in CO_2_-containing syngas mixtures when pulsing NH_3_ or methylamines as basic probe molecules.

## Abstract (web summary)

Methanol synthesis has a high potential for global CO_2_ reduction. Here, the authors identify the oxidation state of the zinc sites on the metallic copper particles as partially positive for an industrial Cu/ZnO/Al_2_O_3_ catalyst under high-pressure reaction conditions.

## Introduction

The high consumption of fossil fuels causes the emission of greenhouse gases and enhances global warming. Therefore, it is the aim of current research to find alternative energy sources and renewable raw materials. One possible alternative is methanol, which is used in a wide range of applications as basic chemical, fuel additive as well as energy carrier^1,2^. It has a high potential to substitute hydrocarbons originating from fossil fuels in many areas^3^. Nowadays, methanol is still industrially produced by the heterogeneously catalysed conversion of syngas, a gas mixture containing CO, CO_2_ and H_2_ obtained from natural gas or coal, over Cu/ZnO/Al_2_O_3_ catalysts at elevated temperatures and pressures^4^. For a methanol production with low CO_2_ footprint, the mixing of exhaust gases from steel production with sustainably produced H_2_ from water electrolysis is a promising route to achieve the gas composition of conventional syngas mixtures^5^. However, many known and unknown impurities in the ppm as well as in the ppb range may be present in such an off-gas-derived syngas, and the effects of most impurities on the catalytic activity, selectivity and stability cannot be predicted with certainty in view of the lack of poisoning studies in literature^6,7^. These impurities can interact as irreversible poisons, which strongly interact with the active sites, or as reversible poisons, which can be desorbed from the active sites and flushed out of the reactor^8,9^. In case of reversible poisoning, the poisons can act as selective probe molecules adsorbing on the active sites without irreversible deactivation. Thus, selective poisoning may allow gaining new insight in the nature of the active sites^10^.

The active site for methanol formation of the industrial Cu/ZnO/Al_2_O_3_ catalyst has been under debate over the last decades. Recent models are the Cu^0^–Zn^0^ surface alloy proposed by the Nakamura and Chorkendorff groups^11,12^, Zn^δ+^ species at the defective Cu^0^ surface claimed by the Schlögl group^13^, graphitic-like ZnO_*x*_ layer on the Cu^0^ particles also suggested by the Schlögl group^14^, and ZnO on the top layer of the Cu^0^ particles postulated by the Rodriguez group^15^. All models are based on strong metal support interactions (SMSI)^16^ resulting in Cu–Zn interface sites that are essential for a highly active catalyst, but they differ in the nature of the Zn species in close contact with Cu^0^, which are controversially debated to be metallic or positively charged. The most active site of methanol synthesis is described as metallic Zn species alloyed into defined Cu^0^ steps^13^. Furthermore, it is proposed that high coverages of oxygen-containing intermediates like formate only exist under industrially relevant high-pressure conditions and lead to a partial oxidation of the metallic Zn sites due to the oxophilic nature of Zn compared with Cu^0^^13^. However, there is no characterisation method available for the Cu/ZnO/Al_2_O_3_ catalyst typically operated above 200 °C and 50 bar^4^, which is able to identify this oxidising effect, because the adjustment of both high-temperature and high-pressure conditions and surface sensitivity is technically impossible for typical single-crystal investigations^11^, transient in situ experiments^17,18^, adsorption and desorption studies^12,19^, in situ infrared (IR) spectroscopy^11,20^, N_2_O frontal chromatography^12,19^, X-ray photoelectron spectroscopy (XPS)^11,12,15,19^, ambient pressure XPS^13^, neutron scattering^13^, microcalorimetry^21,22^, scanning tunnelling microscopy^11^, in situ IR reflection-absorption spectroscopy^11^, high-resolution transmission electron microscopy (TEM)^14,23^, in situ TEM^24^, electron energy loss spectroscopy^14^, in situ X-ray absorption spectroscopy^16^, or operando X-ray diffraction^25^. To confirm the positive charge of the Zn species in close contact with Cu^0^, we establish a surface-sensitive operando method, which allows us to inject selective reversible poisons as probe molecules such as NH_3_ into the syngas feed. By applying this high-pressure pulse unit (HPPU), we are able to identify the nature of the active sites of the working Cu/ZnO/Al_2_O_3_ catalyst under industrially relevant conditions. In detail, we perform conventional methanol synthesis over an industrial Cu/ZnO/Al_2_O_3_ catalyst with CO/CO_2_-containing syngas mixtures above 200 °C and at 60 bar. We investigate the reversible poisoning mechanism of NH_3_ as well as of different types of methylamines and define the corresponding inhibition strength by the non-produced amount of methanol over a defined period of time. Parallel to the reversible poisoning by the amines, we co-feed ethylene into the syngas mixture, which is fast hydrogenated to ethane, we investigate the reversible poisoning mechanism of NO, and we change the syngas mixture from CO_2_- to CO-free gas mixtures to clearly confirm that the methanol synthesis catalyst exposes both highly active Cu^0^–Zn^δ+^ sites and metallic Cu^0^ sites. At the end, the obtained results are discussed in view of the controversial debate on the active site in methanol synthesis demonstrating that the chosen reaction conditions and the time on stream (TOS) control the structure of the catalyst surface.

## Results

### High-pressure pulse experiment (HPPE)

**Fig. 1:**
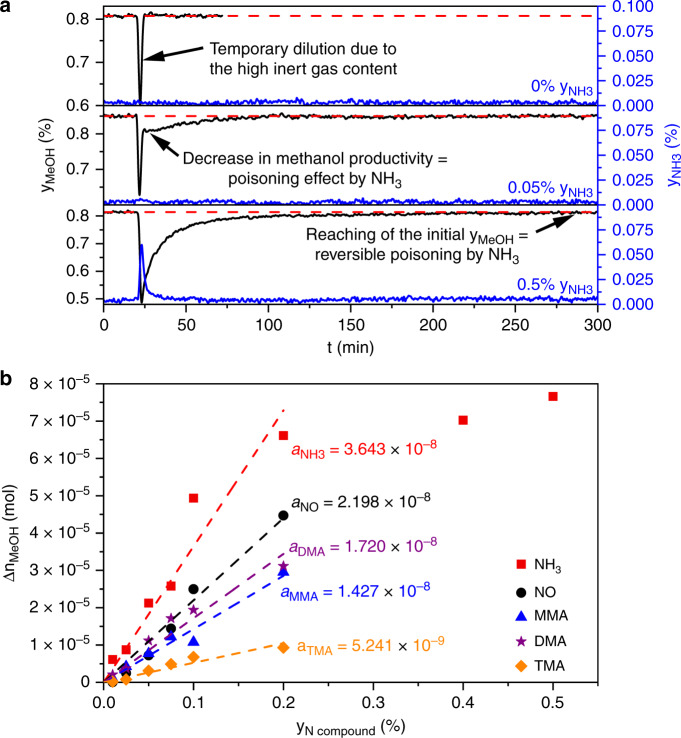
High-pressure pulse experiments and the determined inhibition strengths. **a** Recorded methanol (black curves) and NH_3_ (blue curves) mole fractions during the injection of pulses with different NH_3_ partial pressures over the Cu/ZnO/Al_2_O_3_ catalyst (conditions: 210 °C, 60 bar, 13.5% CO, 3.5% CO_2_, 73.5% H_2_, 9.5% N_2_). **b** Correlation of the determined Δn_MeOH_ (non-produced amount of methanol over a defined period of time) values (Supplementary Note 1) with the injected mole fractions of the investigated N compounds (NH_3_ red squares and line, NO black points and line, monomethylamine (MMA) blue triangles and line, dimethylamine (DMA) violet stars and line, trimethylamine (TMA) orange diamonds and line). The corresponding pulse experiments are shown in Supplementary Figs. 1–5. *a*, the slope of the corresponding linear interpolation, is defined as the inhibition strength.

The self-built HPPU enabled us to inject defined amounts of probe molecules as pulses into the syngas stream under methanol synthesis conditions (210 °C, 60 bar). In this way, a variety of valuable kinetic results can be collected in a short period of time without disturbing the main reaction on the surface sites for a too long period of time to minimize possible irreversible structural transformations. When injecting N-containing compounds acting as reversible poisons, the temporary blocking of active sites only occurs when the poison is present in the feed gas stream, which is exemplarily shown for NH_3_ in Fig. 1a.

A typical HPPE consists of three steps: First, steady state with clean syngas has to be established. The second step is the injection of the probe molecule as a pulse passing through the catalyst bed, and in the third step the period of time is determined until the initial rate of methanol formation is recovered. The pulsing of pure N_2_ (0% NH_3_ in Fig. 1a) illustrates that the used low mole fractions of reversible poisons as well as the high inert content in the sample loop lead to a temporary dilution of the product stream. This sharp negative pulse present in every HPPE is neglected for the interpretation of the results. The rising partial pressure of NH_3_ leads to an increasing loss of methanol activity after the temporary dilution and to longer regeneration times. In addition to the methanol mole fraction (black curves), the NH_3_ mole fractions (blue curves) were also monitored. For the experiment with 0.05% NH_3_, no NH_3_ signal was detected, since it is converted to the methylamines monomethylamine (MMA), dimethylamine (DMA) and trimethylamine (TMA) (Supplementary Fig. 10) reacting with oxygen-containing intermediates of methanol synthesis such as formate^26,27^. This means that the NH_3_ signal of the 0.5% HPPE represents the part of the injected NH_3_, which does not interact with the catalyst surface, because the position of the signal is equal to the temporary dilution. Obviously, the adsorbed NH_3_ species and syngas reactants are part of adsorption equilibria, so that a complete deactivation of methanol synthesis is not possible with a reversible poison in contrast to an irreversible one^8,28^.

In the same way, MMA (Supplementary Fig. 2), DMA (Supplementary Fig. 3) as well as TMA (Supplementary Fig. 4) were also injected as pulses into the feed gas stream under high-pressure conditions to better understand the reversible poisoning mechanism of NH_3_. NO was also used (Supplementary Fig. 5), which is hydrogenated to NH_3_ and H_2_O (Supplementary Fig. 11). All investigated N compounds act as reversible poisons, and for the comparison of the different poisoning strengths the relevant methanol mole fractions were integrated to obtain the non-produced amount of methanol Δ*n*_MeOH_ during the required period of regeneration time (Supplementary Note 1, Supplementary Fig. 6). The correlation of the calculated values Δ*n*_MeOH_ with the different mole fractions of the reversible poisons is shown in Fig. 1b. The Δ*n*_MeOH_ values for every corresponding poison correlate linearly with the mole fraction of the poison for values below 0.2% under the investigated conditions. Above this mole fraction, the poisoning strength reaches a stationary regime due to the limited deactivation effect of reversible poisons, exemplarily shown for NH_3_ (injection of 0.4 and 0.5%). The linear trend of the curves in Fig. 1b can be easily predicted with a one-parameter function through the origin. Here, the slope is defined as the inhibition strength *a*, and the corresponding values are shown in Fig. 1b. When comparing the determined values for the individual inhibition strengths, the following sequence is obtained, in which NH_3_ is the strongest reversible poison and TMA the weakest:

1 $${\mathrm{NH}}_{\mathrm{3}}{\mathrm{ > NO > DMA}} \approx {\mathrm{MMA > TMA}}.$$

**Fig. 2:**
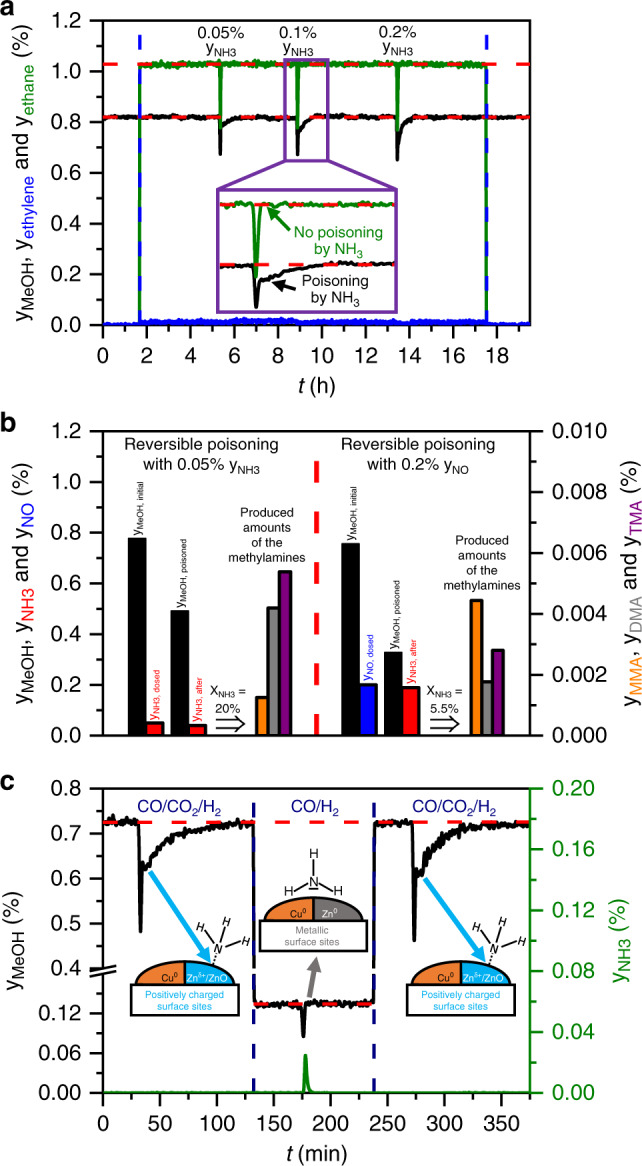
Continuous dosing experiments coupled with HPPEs. **a** Continuous dosing of 1% of ethylene (blue curve), which is hydrogenated to ethane (green curve) under methanol (black curve) synthesis conditions, coupled with HPPEs with NH_3_. **b** Comparison of the continuous dosing of 0.05% NH_3_ with the continuous dosing of 0.2% NO. The black bars describe the initial methanol mole fraction in pure syngas (*y*_MeOH, initial_) and in the presence of the corresponding impurity (*y*_MeOH, poisoned_). The red bars describe the dosed mole fraction of NH_3_ (*y*_NH3, dosed_) and mole fraction in the product gas stream (*y*_NH3, effluent_). The blue bar (y_NO, dosed_) stands for the dosed mole fraction of NO. *X*_NH3_ describes the corresponding degree of NH_3_ conversion to the three methylamines: monomethylamine (orange bar), dimethylamine (grey bar) and trimethylamine (violet bar). **c** Syngas switching experiments from CO/CO_2_/H_2_ to CO/H_2_ and back to CO/CO_2_/H_2_ at 210 °C and 60 bar coupled with HPPEs with NH_3_. Here, the resulting methanol (black curve) and NH_3_ (green curve) mole fractions in the product gas stream are shown. The images visualize the oxidative effect of CO_2_ in the syngas mixture and the corresponding interaction of NH_3_ with the catalyst surface. Orange area = Cu^0^ sites, blue area = Zn^δ+^/ZnO sites, grey area = Zn^0^ sites.

The consecutive reaction of NH_3_ to TMA is one reason for this correlation. NH_3_ requires the highest number of methylation steps until the final product TMA is formed, resulting in the longest blocking of active sites as well as in the highest consumption of oxygen-containing intermediates^26,27^. In contrast, TMA only blocks the active sites by adsorption, since no further methylation is possible (Supplementary Fig. 10b), leading to the shortest residence time on the surface and the lowest inhibition strength. In principle, MMA should be a stronger poison than DMA, but Fig. 1b shows that the inhibition strength of DMA is only slightly higher. Thus, it can be assumed that the methylation steps are fast and a clear distinction between both methylamines is difficult to identify at 210 °C and not observable at 250 °C (Supplementary Fig. 10b). The inhibition strength of NO should also be higher than that of NH_3_ due to the additional hydrogenation step, but the poisoning by NH_3_ and NO proceeds in different ways as explained in Fig. 2.

### Probing the presence of two different active sites

From the first HPPEs, it can be seen that different types of N compounds interact with the active sites of the Cu/ZnO/Al_2_O_3_ catalyst resulting in the observed decrease of methanol formation. Therefore, our work was focused on the identification of the nature of these active sites and on answering the question as to whether other surface sites play an important role. For these investigations, only the probe molecules NH_3_ as reacting N compound (Fig. 2) and TMA as blocking N compound (Supplementary Fig. 12) were considered. The freshly reduced Cu/ZnO/Al_2_O_3_ catalyst has various types of Lewis and Brønsted acid sites with different acid strengths. Actually, the Cu/ZnO/Al_2_O_3_ catalyst developed for industrial reactors is a multi-site system. According to its composition, three different types of adsorption sites can be present^13,29,30^: the Cu–Zn interface, unpromoted metallic Cu^0^ sites, and sites exposed by ZnO. In addition, every type of exposed surface is non-uniform due to the presence of vacancies, steps and kinks^13^. In general, the Cu:Zn ratio as well as the synthesis route is chosen in such a way that an intimate contact between Cu and ZnO is achieved to maximize the number of Cu–Zn interface sites. A significant influence of pure ZnO sites under the conditions of the low-temperature methanol synthesis can be neglected^31^, and the absence of acidic OH groups on the structural promoter Al_2_O_3_ is also excluded due to the absence of acid-catalysed products such as dimethyl ether (Supplementary Note 3)^4^. For Al_2_O_3_, it is generally accepted that it acts as physical spacer to enhance the lifetime as well as the surface area of the Cu/ZnO catalyst maintaining economical methanol production rates over several years^4^. No evidence can be found in literature that Al_2_O_3_ is involved in the reaction mechanism of methanol formation or in the formation of the active sites^32,33^.

The hydrogenation of ethylene and NO was analysed in detail (Fig. 2a, b) to identify the presence of different Cu sites, which has already been claimed in several publications^13,17,29,30^. Figure 2a shows that the continuous dosing of ethylene into the syngas stream leads to its nearly complete hydrogenation to ethane without influencing the rate of methanol formation. Thus, the two reactions are catalysed by two different active sites. Obviously, unpromoted metallic surfaces like Cu^0^ are highly suitable for the hydrogenation of unsaturated hydrocarbons. It is known from literature that Cu^0^ can act as adsorption site for ethylene^34^, and Cu-based catalysts are also applied in other hydrogenation reactions such as the hydrogenation of esters^35^. Furthermore, the high hydrogen content in the syngas is sufficient to ensure that ethylene hydrogenation can proceed without disturbing the formation of methanol on the Cu–Zn interface sites. Adding 0.05, 0.1 and 0.2% of NH_3_ as pulses leads to the expected reversible poisoning of methanol formation, but not to a lowering of the produced amount of ethane neglecting the temporary dilution.

The same result is obtained by comparing the continuous dosing of NH_3_ with the continuous dosing of NO over the Cu/ZnO/Al_2_O_3_ catalyst (Fig. 2b). NO is completely hydrogenated to NH_3_ and H_2_O, and the produced amount of NH_3_ further reacts to methylamines (Supplementary Fig. 11). Figure 2b illustrates that a four times higher content of NO and so of the resulting NH_3_ after hydrogenation compared with the added amount of 0.05% NH_3_ leads to a degree of NH_3_ conversion (*X*_NH3_), which is nearly four times lower: 5.5% compared with 20%. This observation implies that NO is not hydrogenated on the same site where NH_3_ reacts, that is, the NO hydrogenation sites are presumably metallic Cu^0^ as for ethylene. Thus, the observed deactivation of methanol formation by adding NO originates from the re-adsorption of the hydrogenation products NH_3_ and H_2_O, which is favoured under the applied high-pressure conditions according to the following consecutive reaction sequence^36^

2 $${\mathrm{NO}}\mathop { \to }\limits^{ + {\mathrm{H}}_2} {\mathrm{NH}}_3\mathop { \to }\limits^{ + {\mathrm{CH}}_3{\mathrm{OH}}} {\mathrm{methyl}}\,{\mathrm{amines}}.$$

As a result, it seems that NH_3_ does not prefer unpromoted metallic Cu^0^ as adsorption site, which was also the outcome of NH_3_ adsorption studies on Cu^0^ single crystals^37,38^. To confirm this hypothesis, the syngas mixture was switched from CO/CO_2_/H_2_ to CO/H_2_ and back to the CO_2_-containing feed at constant reaction temperature and pressure, which is shown in Fig. 2c. The absence of CO_2_ in the syngas mixture results in a strongly reducing atmosphere, which induces the formation of metallic surfaces on the Cu/ZnO/Al_2_O_3_ catalyst. Increasing reaction temperatures and longer reaction times are known to result in brass formation^16^. Here, the lower production rates for methanol originate from a changed mechanism of methanol formation from the formate pathway to the formyl pathway, which is kinetically not preferred on Zn-promoted Cu^0^ surfaces^17^. The adsorbates of the formyl mechanism such as CO_ads_ and HCO_ads_ bind through their C atoms to the catalyst surface, and no oxidising effect is induced due to the absence of CO_2_ and H_2_O in contrast to the formate pathway. Therefore, the combination of this experiment with HPPEs shows no reversible poisoning and no conversion of NH_3_ in the CO/H_2_ mixture. Thus, the composition of the syngas mixture controls the oxidation state of the catalyst surface, which can be changed reversibly under low-temperature conditions as confirmed in many studies^11,16,24^. In summary, all experiments shown in Fig. 2 indicate that metallic Cu^0^ or Cu^0^–Zn^0^ sites are not the adsorption sites for NH_3_, implying that the observed reversible NH_3_ poisoning in the presence of CO_2_ must selectively proceed on positively charged Cu^0^–Zn^δ+^ interface sites due to the oxidising effect of the oxygen-containing adsorbates.

### The role of CO_2_

**Fig. 3:**
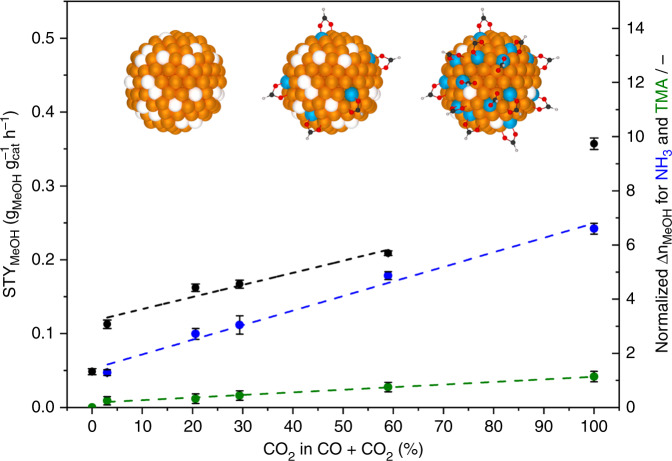
Influence of CO_2_ on the methanol productivity and on the oxidation state of the catalyst surface. Correlation of the methanol productivity (black points and line) and of the normalized Δ*n*_MeOH_ values (Supplementary Note 2) of NH_3_ (blue points and line) and TMA (green points and line) as a function of the CO_2_ content in the syngas from pure CO hydrogenation (0%) to pure CO_2_ hydrogenation (100%). Illustrations: Metallic Cu^0^ particles (orange balls), metallic Zn^0^ species (white balls), positively charged Zn species (blue balls) and formate as adsorbates. The error bars were determined by measuring every point five times.

It was already shown that adsorbates like formate or other oxygen-containing species originating from CO_2_ play not only a crucial role in the methylation of amines, but also in the partial oxidation of Zn species in contact with metallic Cu^0^ sites^12,13^. This type of oxidation is favoured for Zn due to its higher oxophilicity compared with Cu^13,17^. Providing evidence for this hypothesis requires that the corresponding investigations must be performed under high-pressure methanol synthesis conditions to generate the necessary adsorbates on the surface. For methanol synthesis over Cu/ZnO/Al_2_O_3_, the high-pressure pulse method combined with selective poisons as probe molecules enables us to analyse the oxidation effect under working conditions. For the investigation of the adsorbate-induced surface oxidation, the CO_2_ content in the feed gas was increased from 0 (CO hydrogenation) to 100% (CO_2_ hydrogenation) to generate different coverage degrees of the reaction intermediates, and for every gas mixture the normalized Δ*n*_MeOH_ values (Supplementary Note 2) were determined by pulsing NH_3_ and TMA (Fig. 3).

Adding small amounts of CO_2_ in the syngas feed results in a strong increase of the methanol production rate, which further increases with rising CO_2_ content, because CO_2_ is much faster hydrogenated to methanol than CO^17^. In general, it is accepted that the highest production rate is achieved at low CO_2_ mole fractions around 2–4%^4^. This statement is only valid for high degrees of syngas conversion and high methanol and H_2_O mole fractions (Supplementary Fig. 13). In the differential kinetic regime, product inhibition effects by H_2_O are not significant enough to lower the rate of methanol formation strongly^17,36^.

In addition to the increasing production rate of methanol, Fig. 3 illustrates that the observed poisoning strengths of NH_3_ and TMA increase linearly as a function of the CO_2_ content in the syngas mixture. Therefore, higher CO_2_ mole fractions lead to increasing coverages of the resulting oxygen-containing adsorbates exemplary shown as formate, and these adsorbates create more Cu^0^–Zn^δ+^ interface sites. In this way, the adsorption capacity of TMA is enhanced, and more NH_3_ molecules can be adsorbed and further converted.

On the basis of the presented results and of different literature reports, we assume that the initial reduction of the catalyst surface induces the migration of metallic Zn species onto the metallic Cu^0^ particles forming the Cu^0^–Zn^0^ surface alloy^11^. This state of the surface seems to be stable under the conditions of CO hydrogenation, but not in the presence of CO_2_, which leads to the oxidation of the metallic Zn species forming Zn^δ+^ species on the Cu^0^ surface^13^. It cannot be clarified if all Zn sites in contact with Cu^0^ are oxidized in the CO_2_/H_2_ gas mixture, but the majority of the sites should have a positively charged nature. The models of the active site of the Cu/ZnO/Al_2_O_3_ catalyst of the Nakamura and Schlögl groups are rather similar proposing a Cu^0^–Zn^0^ surface alloy^11^ and Cu steps with Zn alloyed into it^13^, respectively. Behrens et al.^13^ assumed the partial oxidation of the metallic Zn species, which cannot be neglected under industrially relevant coverage degrees. In contrast, the assumptions of the Nakamura and Chorkendorff groups^11,12,19^ are mainly based on results obtained under atmospheric pressure or ultra-high vacuum, which result in low degrees of conversion and coverages. Thus, the fraction of oxidized Zn sites compared to the metallic ones is small, and the oxidation effect is hardly observable. For example, most characterization methods like our pulse experiments at 1 bar (Supplementary Fig. 9d) provide an averaged result over the whole Cu surface area, which is mainly metallic. However, the Cu^0^–Zn^0^ surface alloy model^11^ provides a highly suitable description of the catalyst surface after reduction and presumably for methanol synthesis from CO.

## Discussion

We analysed the rate of methanol formation reaction mainly from CO/CO_2_-containing syngas mixtures over an industrial Cu/ZnO/Al_2_O_3_ catalyst, which can be reversibly deactivated by NH_3_, which initializes the consecutive methylation reactions. The step-by-step methylation with presumably oxygen-containing intermediates like formate seems to be the major factor determining the inhibition strength of the amines, because NH_3_ is the strongest inhibitor. Both reactions selectively take place on the positively charged Zn species, which are formed by the diffusion of ZnO_*x*_ species onto the metallic Cu^0^ particles creating additional interface sites. The unpromoted Cu^0^ sites act as hydrogenation sites for ethylene as well as NO, and sites exposed by ZnO nanoparticles are not active under the low-temperature conditions. For the hydrogenation products of NO, the re-adsorption of NH_3_ and H_2_O is favoured under the high-pressure conditions. In addition, we showed that the CO_2_ content in the syngas is essential to achieve the optimum oxidation state of the interface sites, which are less active and more metallic in the absence of CO_2_. The resulting adsorbate-induced oxidation of surface sites by formate and the importance of Lewis acids on metallic Cu^0^ for the formation of methanol were also observed in recent studies^39,40^.

**Fig. 4:**
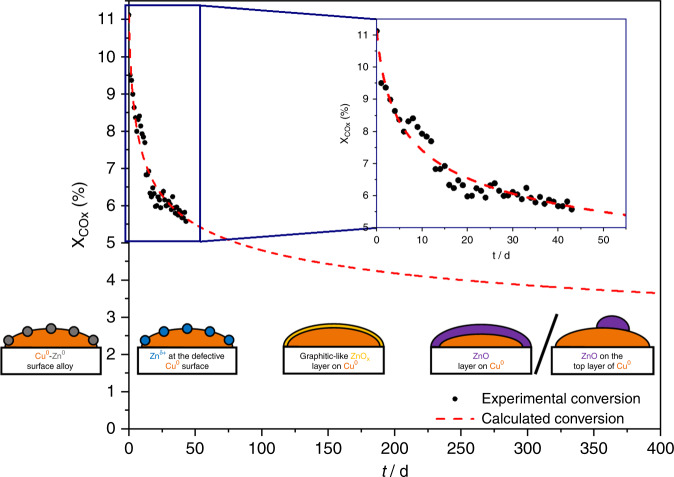
Long-term methanol synthesis over the industrial Cu/ZnO/Al_2_O_3_ catalyst at 210 °C and 60 bar. Recorded degrees of conversion (black points) under differential controlled conditions. The dashed red curve describes the intra- and extrapolation of the experimental data, which was calculated with the MATLAB^®^ software according to the studies of Fichtl et al.^18^. Illustrations from right to left: Cu^0^–Zn^0^ surface alloy according to Nakamura et al.^11^. Zn^δ+^ species at the defective Cu^0^ surface according to Behrens et al.^13^, graphitic-like ZnO_*x*_ layer on Cu^0^ according to Lunkenbein et al.^14^. ZnO layer on Cu^0^ according to Lunkenbein et al.^23^ as well as Fichtl et al.^18^ and ZnO on the top layer of Cu^0^ according to Kattel et al.^15^.

Actually, the statement that CO_2_ is important for the oxidation state of the surface goes back to Klier et al.^41^, who assumed erroneously that Cu^+^ is the active site. Figure 4 illustrates that all newer models can be applied to describe the structural evolution of the Cu/ZnO/Al_2_O_3_ catalyst as a function of TOS.

The strong loss of ~50% of the initial activity in the first weeks is inevitable for Cu-based catalysts developed for industrial reactors due to thermal sintering and restructuring^4^. Therefore, the Cu/ZnO/Al_2_O_3_ catalyst is a dynamic system with a continuously changing structure, so that true steady state for a couple of days cannot be reached even under industrial timescales as illustrated by the extrapolated curve. We propose the following structural changes as a function of TOS from left to right in Fig. 4: At the beginning, highly reduced ZnO_*x*_ species migrate onto the metallic Cu^0^ nanoparticles during reduction forming a finely dispersed Cu^0^–Zn^0^ alloy according to Nakamura et al.^11^. The change to a CO_2_-containing syngas mixture initializes the formation of different oxygen-containing adsorbates as well as the oxidation of the metallic Zn species to Zn^δ+^ species at the defective Cu^0^ surface according to Behrens et al.^13^. The migration of further Zn species results in the formation first of a graphitic-like ZnO_*x*_ layer on Cu^0^ according to Lunkenbein et al.^14^ and then a more crystalline and stable thick ZnO layer or particles according to Kattel et al.^15^. The formation of thick ZnO layers due to segregation from the metallic Cu^0^ phase were observed in recent long-term studies^18,23^ clarifying that the SMSI process is thermodynamically preferred, but kinetically slow. Under industrially relevant conditions our results clearly confirm the presence of highly active Cu^0^–Zn^δ+^ interface sites embedded in the structurally constantly changing matrix provided by the Cu/ZnO/Al_2_O_3_ catalyst Thus, by applying our developed high-pressure pulse method, we were able to identify the nature of these interface sites, which has not been possible by other characterization methods lacking the combination of surface sensitivity and industrially relevant reaction conditions.

## Methods

### Activation and aging of the Cu/ZnO/Al_2_O_3_ catalyst

All measurements in this work were performed with the same industrial Cu/ZnO/Al_2_O_3_ catalyst provided by Clariant Produkte (Deutschland) GmbH using a standard high-pressure flow set-up including mass flow controllers (MFC, Bronkhorst Deutschland Nord GmbH) for the adjustment of the necessary volume flows, shutdown (Swagelok Company) and multi-port (VICI Valco Instruments) valves, one tubular 1/4-inch stainless-steel reactor mounted in a 1-zone oven (HTM Reetz GmbH) and equipped with an internal thermocouple in order to achieve the desired reaction temperature in the catalyst bed, a back pressure regulator (BPR, Equilibar Precision Pressure Control) connected to a process pressure controller (PC, Bronkhorst Deutschland Nord GmbH) for the reaching of high-pressure conditions (up to 60 bar) in the reactor and a unique self-built HPPU. In addition, all stainless-steel tubes and parts, which were contaminated by the corrosive reversible poisons used in this work, were coated with SilcoNert 2000® provided by SilcoTek GmbH and heated up to 150 °C to minimize any kind of adsorption phenomena. For the quantification of the product gas streams, a combination of a FTIR spectrometer (Nicolet is50, Thermo Fisher Scientific) and a Micro GC device (Micro GC 490, Agilent) was used. The spectrometer contains a transmission gas cell in the sample holder with ZnSe windows, a pathlength of 150 mm and a cell volume of 7.5 ml. In addition, the spectrometer offers two different types of detectors for recording IR spectra: The thermal DTGS (deuterated triglycine sulfate) and the liquid nitrogen cooled MCT (mercury cadmium telluride) detectors. In the case of the Micro GC device, a pump, a micro-machined injector with an injection volume of 5 µl, a Molsieve 5A column and a micro-machined thermal conductivity detector were used for recording the chromatograms. The calibrations of both analytics were validated and the carbon balance (C-balance) of every kinetic experiment performed in this work was in the acceptable range of 100% ± 1%.

3 $${\mathrm{C}} - {\mathrm{balance}} = \frac{{y_{C,\mathrm{out}}}}{{y_{C,\mathrm{in}}}} = \frac{{y_{\mathrm{CO},\mathrm{out}} + y_{\mathrm{CO2},\mathrm{out}} + y_{\mathrm{MeOH},\mathrm{out}}}}{{y_{\mathrm{CO},\mathrm{in}} + y_{\mathrm{CO2},\mathrm{in}}}}.$$

Here, the ratio between the mole fraction of all carbon-containing compounds (CO, CO_2_, CH_3_OH) in the product gas stream *y*_*c,*out_ and the mole fraction of all carbon-containing compounds (CO, CO_2_) in the feed *y*_*c,*in_ yields the C-balance.

For the reduction of the catalyst precursor, the temperature was increased to 175 °C with a heating rate of 1 °C min^−1^ using a gas flow rate of 500 Nml min^−1^ g_cat_^−1^ diluted H_2_ (2% H_2_ (99.999%) in N_2_ (99.999%)) for 15 h. In a second step, the temperature was increased to 240 °C with 1 °C min^−1^ and held for 30 min. After the second increase of the temperature, the reduced catalyst was ready for methanol synthesis.

To reach steady-state conditions in a relatively short period of time, the catalyst was aged before performing the kinetic experiments. The reaction temperature was increased to 250 °C at 60 bar to establish equilibrium-controlled conditions ensuring a reproducible deactivation by exposing the whole catalyst bed to the same gas composition at high degrees of conversion according to the study by Fichtl et al.^18^. As standard syngas mixture, the following composition was used: 13.5% CO (99.997%), 3.5% CO_2_ (99.998%), 73.5% H_2_ (99.999%) and 9.5% N_2_ (99.999%). All measurements in this study were performed in the differential regime with the necessary syngas flow rate.

### High-pressure pulse experiment (HPPE)

**Fig. 5:**
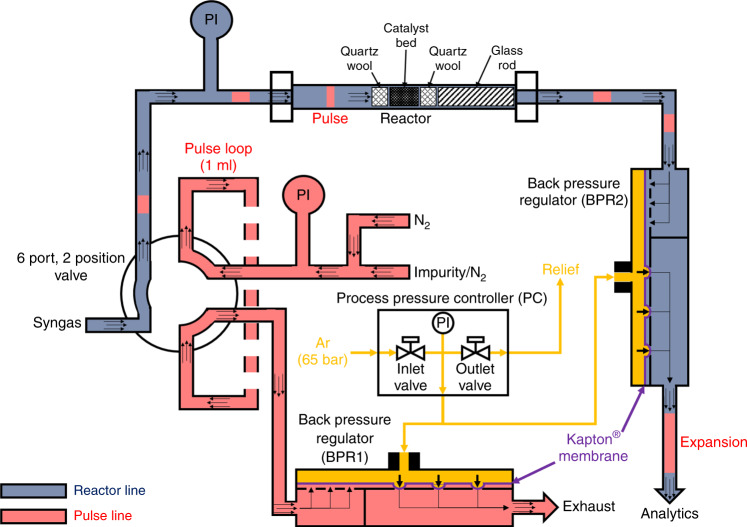
Scheme of the high-pressure pulse unit integrated in the flow set-up. PI is defined as pressure sensor in the corresponding line. The area in blue describes the flow direction of the syngas mixture, the red area of the injected impurities and the orange area of the reference gas (Ar) for pressure regulation.

For the HPPE, a self-built HPPU was developed to inject probe molecules as pulses under high-pressure conditions, which is schematically shown in Fig. 5.

The HPPU consists of one 6 port, 2 position valve equipped with a 1 ml sample loop and two identical back pressure regulators (BPR1 and BPR2) coupled with one process PC. BPR2 is integrated in the reactor line between the reactor outlet and the analytics and BPR1 in the pulse line between the sample loop and the exhaust. The method for the injection of a pulse over the catalyst bed in the reactor under high-pressure conditions is as follows: The BPRs consist of two parts, which are separated from each other by a Kapton^®^ membrane. The upper parts are connected with PC and the other parts with the incoming gas flow from the reactor or the sample loop on the inlet sites and on the outlet sites with the analytics or exhaust. The task of the PC is to reach the same pressure level in both back pressure regulators. It consists of two valves and a pressure sensor PI. For the setting of pressure values up to 60 bar, 65 bar of Ar (99.999%) must be connected on the inlet valve. When a pressure value is set in the program of the PC, the inlet as well as outlet valve is opened and closed until the pressure sensor measures the given value. Consequently, the pressure in both upper parts of BPR1 and BPR2 increases and so the membranes are pressed down. At this moment, the gas flows are stopped and the pressure in the reactor line and pulse line builds up until the same pressure level is reached like in the upper parts. Then, the membranes are lifted up to the starting positions and the gas mixtures can flow again. If both pressure sensors PI in the reactor and pulse line show the same pressure value, then the valve can be switched to inject the first pulse into the syngas stream.

**Fig. 6:**
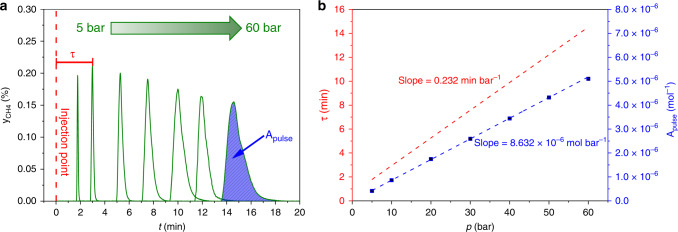
Validation of the self-built high-pressure pulse unit with inert CH_4_ pulses over α-Al_2_O_3_. **a** Recorded methane pulses (green curves) injected over α-Al_2_O_3_ as inert bed at different pressures from 5 to 60 bar. *τ* is defined as the retention time of the CH_4_ pulses. **b** Correlation of the determined retention times *τ* (red points and line) and pulse areas *A*_pulse_ (blue points and line) against the set pressure levels. The error bars were determined by measuring every point 5 times.

The results of the validation are shown in Fig. 6. Here, 200 mg of α-Al_2_O_3_ (sieve fraction: 250–355 µm, Südchemie GmbH) were placed in the isothermal zone of the reactor and fixed between two plugs of quartz wool to simulate an inert catalyst bed. One glass rod was placed after the bed to lower the dead volume. Then, 0.3% CH_4_ (99.995%) in N_2_ (99.999%) as IR-active, but inert gas was injected as pulses into a 50 Nml min^−1^ N_2_ (99.999%) flow passing through the α-Al_2_O_3_ bed at 5, 10, 20, 30, 40, 50 and 60 bar. The retention time *τ* and the pulse area *A*_pulse_ of the recorded pulses (Fig. 6a) were determined and correlated with the set pressures (Fig. 6b).

The dashed red line in Fig. 6a represents the injection point of the pulses at time 0. Increasing the pressure leads to longer *τ* and increasing *A*_pulse_. In this case, only the blue curve (*A*_pulse_) in Fig. 6b goes through the origin and not the red curve (*τ*). The reason for this is the constant operation of the analytics under atmospheric pressure and so the expansion of the pulses after the BPR2 (Fig. 5). In general, the volume flow $\dot V$ of gaseous compounds is reduced by increasing the pressure level *p* according to the ideal gas law (Eq. (4)), since the molar flow $\dot n$ (due to a constant mass flow provided by the used MFC at every pressure level), the gas constant *R* and the temperature *T* (due to small changes in the pressure level) can be seen as constant:

4 $$p = \frac{{\dot n \cdot R \cdot T}}{{\dot V}} = {\mathrm{const}}. \cdot \frac{1}{{\dot V}}$$

5 $$\tau = \frac{V}{{\dot V}} = {\mathrm{const}}. \cdot p$$

In contrast, the resulting retention time or residence time *τ*, which is defined as reactor volume *V* divided by the volume flow $\dot V$ of the pulses is proportional to the applied pressure. The corresponding value for the constant in Eq. (5) is equal to the slope *m* of the lines in Fig. 6b.

The linear behaviour of both functions (Fig. 6b), the absence of steps in the recorded pulses and the acceptable tailing at higher pressures (Fig. 6a) demonstrate a successful validation of the HPPU. Consequently, significant pressure drops and backmixing of the probe molecules can be excluded. In addition, the small error bars (every measurement was repeated five times) in Fig. 6b underline the reproducibility of the pulse method.

For a typical HPPE, 100 mg of the industrial Cu/ZnO/Al_2_O_3_ catalyst (sieve fraction 250–355 µm) were placed in the tubular reactor. In the first step, the initial methanol mole fraction in the standard syngas mixture at 210 °C and 60 bar was recorded under differential controlled conditions. Then, the different N compounds were injected as pulses from the corresponding gas cylinder: 0.5% NH_3_ (99.999%) in N_2_ (99.999%), 0.2% NO (99.5%) in N_2_ (99.999%), 0.2% monomethylamine (MMA, 99.5%) in N_2_ (99.999%), 0.2% dimethylamine (DMA, 99.5%) in N_2_ (99.999%), 0.2% trimethylamine (TMA, 99.5%) in N_2_ (99.999%). The mole fraction in the sample loop was set at 0, 0.01, 0.025, 0.05, 0.075, 0.1 and 0.2%. The measurement with 0% corresponds to a pulse with pure inert gas N_2_ (99.999%). In the case of NH_3_, additional HPPEs were performed with 0.4 and 0.5%. In addition, 0.05, 0.1 and 0.2% NH_3_ were injected at 230 and 250 °C at constant 60 bar as well as at 1, 10, 30 and 60 bar at constant 210 °C.

### Co-feeding of NH_3_, NO, MMA, DMA and TMA

The continuous dosing experiments with various N compounds were performed over 200 mg (sieve fraction 250–355 µm) of the industrial methanol catalyst. For the dosing of 0.05% NH_3_ (from 0.5% NH_3_ (99.999%) in N_2_ (99.999%)) and 0.2% NO (from 2% NO (99.5%) in N_2_ (99.999%)), the reaction temperature and pressure were set at 210 °C and 60 bar. In the case of dosing of 0.051% NH_3_, 0.0153% MMA (from 0.2% MMA (99.5%) in N_2_ (99.999%)), 0.0170% DMA (from 0.2% DMA (99.5%) in N_2_ (99.999%)) and 0.0157% TMA (from 0.2% TMA (99.5%) in N_2_ (99.999%)), the reaction temperature was increased to 250 °C. After achieving a constant initial methanol mole fraction applying the standard syngas mixture, the corresponding N compound was continuous dosed over the catalyst bed for a defined period of time by replacing the internal standard N_2_ in the standard syngas mixture. At the end, the gas stream of the N compounds was switched off to measure the methanol activity in pure syngas.

### Co-feeding of ethylene coupled with HPPE with NH_3_/TMA

The combination of methanol synthesis in the presence of ethylene coupled with HPPEs with NH_3_ and TMA were performed with 100 mg of the industrial Cu/ZnO/Al_2_O_3_ catalyst (sieve fraction 250–355 µm) mounted in the tubular reactor. In the first step, the initial methanol mole fraction was determined with the standard syngas mixture at 210 °C and 60 bar. Then, 1% of the internal standard N_2_ was replaced by ethylene (from 20% ethylene (99.995%) in N_2_ (99.999%)) and after a defined period of time, the feed was switched back to the standard syngas mixture. During the adding of ethylene, 0.05%, 0.1% and 0.2% of NH_3_ (from 0.5% NH_3_ (99.999%) in N_2_ (99.999%)) and TMA (from 0.2% TMA (99.5%) in N_2_ (99.999%)) were injected as pulses.

### Syngas switching experiments coupled with HPPE with NH_3_/TMA

The combination of syngas switching experiments under methanol synthesis conditions with HPPEs with NH_3_ and TMA were carried out over 100 mg of the industrial Cu/ZnO/Al_2_O_3_ catalyst (sieve fraction 250–355 µm). Here, the syngas feed was changed from the standard mixture containing CO/CO_2_ to 17% CO (99.997%), 73.5% H_2_ (99.999%), 9.5% N_2_ (99.999%) and back to the standard mixture. At each instant, methanol was produced at 210 °C and 60 bar. In addition, pulses containing 0.2% of NH_3_ (from 0.5% NH_3_ (99.999%) in N_2_ (99.999%)) and TMA (from 0.2% TMA (99.5%) in N_2_ (99.999%)) were injected into the different feed gas compositions.

### CO/CO_2_ variation experiment coupled with HPPE with NH_3_/TMA

The combination of CO/CO_2_ variation experiments under methanol synthesis conditions with HPPEs with NH_3_ and TMA were performed with 100 mg of the Cu/ZnO/Al_2_O_3_ catalyst (sieve fraction 250–355 µm) in the tubular reactor. The reaction temperature and pressure were constant at 210 °C and 60 bar and the CO_2_ content in the syngas feed was changed from 0 to 17% (0, 0.5, 3.5, 5, 10, 17%) by substituting or adding CO to maintain the carbon mole fraction at constant 17% (CO + CO_2_). For every new syngas mixture, the methanol productivity was recorded and 0.2% NH_3_ (from 0.5% NH_3_ (99.999%) in N_2_ (99.999%)) as well as TMA (from 0.2% TMA (99.5%) in N_2_ (99.999%)) were injected as pulses. Every measurement was repeated five times to determine the corresponding error bars.

### Long-term measurement

**Fig. 7:**
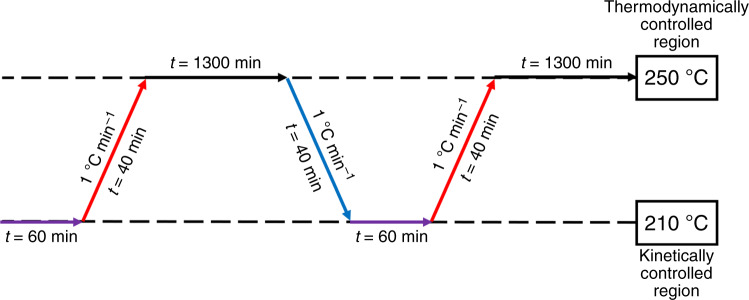
Experimental protocol of the long-term measurement adapted from the studies by Fichtl et al.^18^. The violet arrow describes the period of time to record the kinetic data point, the red arrow describes the heating rate, the black arrow describes the aging of the catalyst bed under equilibrium conditions and the blue arrow describes the cooling rate.

The procedure of the long-term measurement was adapted from Fichtl et al.^18^. and is shown in Fig. 7.

Three hundred milligrams of the industrial methanol synthesis catalyst (sieve fraction 250–355 µm) were mixed with 1800 mg purified α-Al_2_O_3_ (sieve fraction 510–750 µm) and placed in the tubular reactor. The high dilution should prevent the formation of hot spots, so that a homogeneous temperature profile over the entire catalyst bed was obtained. After the standard activation procedure, the gas stream was changed to the standard syngas mixture and the reaction pressure was increased up to 60 bar. The initial activity test was performed at 210 °C for 60 min under kinetically controlled conditions. Then, the catalyst was heated to the aging temperature of 250 °C and the volume flow was reduced to reach the equilibrium conversion. The equilibrium condition was held for 90% of a day. The decrease of the reaction temperature to 210 °C and the increase of the flow rate allowed to measure the next kinetically controlled rate of methanol formation. The continuous change between the two conditions was carried out until a total TOS of 6 weeks was reached. Besides, the recorded experimental curve was interpolated by using the following empirical power-law model (PLM):

6 $$\frac{{\mathrm{d}a_{\mathrm{rel}}}}{{\mathrm{d}t}} = - k_d \cdot a_{\mathrm{rel}}\left( t \right)^m,$$

7 $$a_{\mathrm{rel}} = \frac{{X_{\mathrm{CO}x}(\mathrm{TOS})}}{{X_{\mathrm{CO}x}(\mathrm{TOS} = 0)}},$$

*a*_rel_ is defined as the relative activity, the ratio between the time-dependent CO_*x*_ (CO + CO_2_) conversion (*X*_CO*x*_*(*TOS*)*) and the CO_*x*_ conversion of the initial activity (*X*_CO*x*_(TOS = 0)). *k*_*d*_ is the deactivation rate constant and *m* is an empirical factor or the reaction order. For the calculation of the theoretical curve, the MATLAB^®^ software was used and the procedure was adapted from the work of Fichtl et al.^18^.

## Supplementary information

Supplementary Information

Peer Review File

## Data Availability

The data sets generated during and/or analysed during the current study are available from the corresponding author on reasonable request.

## References

[1] Sehested J (2019). Industrial and scientific directions of methanol catalyst development. J. Catal..

[2] Alvarado, M. *4th IMPCA Mississippi Conf. America* (New Orleans, 2016).

[3] Olah GA (2006). Beyond oil and gas. The methanol economy. Angew. Chem. Int. Ed..

[4] Hansen, J. B. et al. *Handbook of Heterogenous Catalysis: Methanol Synthesis* Ch. 13.13 (Wiley-VCH, Weinheim, 2008).

[5] Schittkowski J (2018). Methanol synthesis from steel mill exhaust gases. Challenges for the industrial Cu/ZnO/Al_2_O_3_ catalyst. Chem. Ing. Tech..

[6] Spencer MS (2005). Metal catalyst design and preparation in control of deactivation. Annu. Rev. Mater. Res..

[7] Remus, R. et al. Best Available Techniques (BAT). Reference Document for Iron and Steel Production: Industrial Emissions Directive 2010/75/ EU (Integrated Pollution Prevention and Control). *JRC Reference Report, European Commission, Joint Research Centre, Institute for Prospective Technological Studies* (Seville, 2013).

[8] Bartholomew CH (2001). Mechanisms of catalyst deactivation. Appl. Catal. A: Gen..

[9] Moulijn, J. A. et al. *Handbook of Heterogenous Catalysis: Deactivation and Regeneration* Ch. 7.1 (Wiley-VCH, Weinheim, 2008).

[10] Dahl S (1999). Role of steps in N_2_ activation on Ru(0001). Phy. Rev. Lett..

[11] Nakamura J (2003). On the issue of the active site and the role of ZnO in Cu/ZnO methanol synthesis catalysts. Top. Catal..

[12] Kuld S (2016). Quantifying the promotion of Cu catalysts by ZnO for methanol synthesis. Science.

[13] Behrens M (2012). The active site of methanol synthesis over Cu/ZnO/Al_2_O_3_ industrial catalysts. Science.

[14] Lunkenbein T (2015). Formation of a ZnO overlayer in industrial Cu/ZnO/Al_2_O_3_ catalysts induced by strong metal-support interactions. Angew. Chem..

[15] Kattel S (2017). Active sites for CO_2_ hydrogenation to methanol on Cu/ZnO catalysts. Science.

[16] Grunwaldt J-D (2000). In situ investigations of structural changes in Cu/ZnO catalysts. J. Catal..

[17] Studt F (2015). The mechanism of CO and CO_2_ hydrogenation to methanol over Cu-based catalysts. ChemCatChem.

[18] Fichtl MB (2015). Kinetics of deactivation on Cu/ZnO/Al_2_O_3_ methanol synthesis catalysts. Appl. Catal. A: Gen..

[19] Kuld S (2014). Quantification of zinc atoms in a surface alloy on copper in an industrial-type methanol synthesis catalyst. Angew. Chem..

[20] Askgaard TS (1995). A kinetic model of methanol synthesis. J. Catal..

[21] Naumann d’Alnoncourt R (2003). The influence of ZnO on the differential heat of adsorption of CO on Cu catalysts: a microcalorimetric study. J. Catal..

[22] Naumann d’Alnoncourt R (2006). The influence of strongly reducing conditions on strong metal–support interactions in Cu/ZnO catalysts used for methanol synthesis. Phys. Chem. Chem. Phys..

[23] Lunkenbein T (2016). Bridging the time gap. A copper/zinc oxide/aluminum oxide catalyst for methanol synthesis studied under industrially relevant conditions and time scales. Angew. Chem. Int. Ed..

[24] Hansen PL (2002). Atom-resolved imaging of dynamic shape changes in supported copper nanocrystals. Science.

[25] Martin O (2016). Operando synchrotron X-ray powder diffraction and modulated-excitation infrared spectroscopy elucidate the CO_2_ promotion on a commercial methanol synthesis catalyst. Angew. Chem..

[26] Vedage GA (1985). Chemical trapping of surface intermediates in methanol synthesis by amines. J. Catal..

[27] Gredig SV (1995). Synthesis of methylamines from carbon dioxide and ammonia. J. Chem. Soc., Chem. Commun..

[28] Twigg MV (2003). Deactivation of copper metal catalysts for methanol decomposition, methanol steam reforming and methanol. Synth. Top. Catal..

[29] Zander S (2013). The role of the oxide component in the development of copper composite catalysts for methanol synthesis. Angew. Chem. Int. Ed..

[30] Kunkes EL (2015). Hydrogenation of CO_2_ to methanol and CO on Cu/ZnO/Al_2_O_3_. Is there a common intermediate or not?. J. Catal..

[31] Song H (2017). Spinel-structured ZnCr_2_O_4_ with excess Zn is the active ZnO/Cr_2_O_3_ catalyst for high-temperature methanol synthesis. ACS Catal..

[32] Saito M (1996). Development of copper/zinc oxide-based multicomponent catalysts for methanol synthesis from carbon dioxide and hydrogen. Appl. Catal. A: Gen..

[33] Kurtz M (2003). Deactivation of supported copper catalysts for methanol synthesis. Catal. Lett..

[34] Franken PE (1975). Ethylene adsorption on thin films of Ni, Pd, Pt, Cu, Au and Al; work function measurements. Surf. Sci..

[35] Li F (2017). Selective hydrogenation of ethylene carbonate to methanol and ethylene glycol over Cu/SiO_2_ catalysts prepared by ammonia evaporation method. Int. J. Hydrog. Energy.

[36] Sahibzada M (1998). Methanol synthesis from CO/CO_2_/H_2_ over Cu/ZnO/Al_2_O_3_ at differential and finite conversions. J. Catal..

[37] Biemolt W (1992). The adsorption site of ammonia at copper surfaces. Catal. Today.

[38] van de Kerkhof GJ (1993). Dissociation of ammonia on a copper surface and the effect of oxygen coadsorption: a quantum-chemical study. Surf. Sci..

[39] Matsubu JC (2017). Adsorbate-mediated strong metal-support interactions in oxide-supported Rh catalysts. Nat. Chem..

[40] Kim J (2019). Surface Lewis acidity of periphery oxide species as a general kinetic descriptor for CO_2_ hydrogenation to methanol on supported copper nanoparticles. ACS Catal..

[41] Klier K (1982). Catalytic synthesis of methanol from COH_2_: IV. The effects of carbon dioxide. J. Catal..

