# Supplementary material for: Identifying the nature of the active sites in methanol synthesis over Cu/ZnO/Al2O3 catalysts
Source: Nat Commun. 2020 Aug 4;11:3898. doi: 10.1038/s41467-020-17631-5 (PMC7403733; doi:10.1038/s41467-020-17631-5)
Supplement: Supplementary file 1 — Supplementary Information [file 41467_2020_17631_MOESM1_ESM.pdf]

Identifying the nature of the active sites in methanol synthesis over $Cu/ZnO/Al_2O_3$ catalysts

Supplementary Information (SI)

Daniel Laudenschleger[1], Holger Ruland[2], Martin Muhler*[1,2]

[1]Laboratory of Industrial Chemistry, Ruhr University Bochum, Universitätsstraße 150, D-44780 Bochum, Germany
E-Mail: muhler@techem.rub.de
[2]Max Planck Institute for Chemical Energy Conversion, Stiftstraße 34-36, D-45470 Mülheim an der Ruhr, Germany

Table of Contents

Supplementary Figures

Supplementary Notes

Supplementary Figures

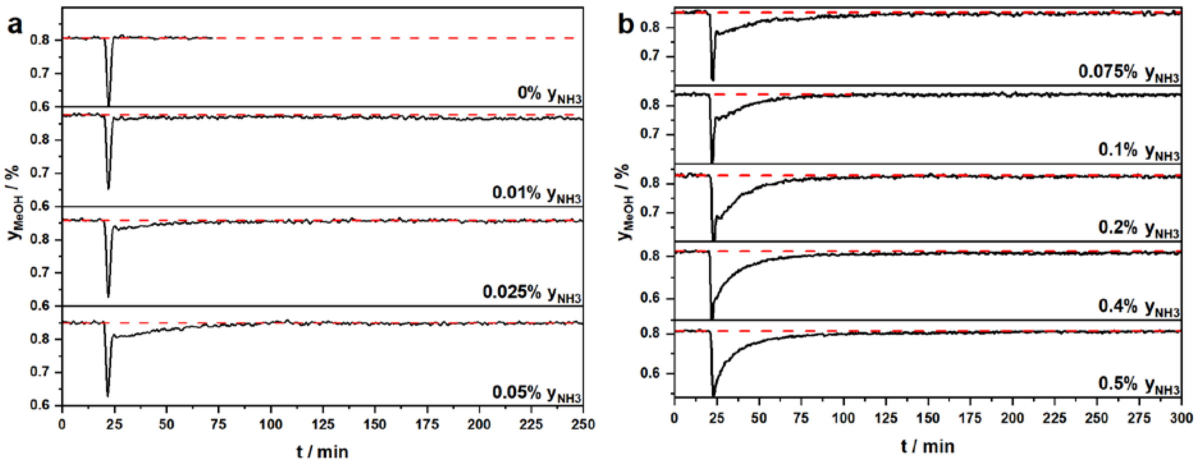

Supplementary Figure 1. HPPEs with NH_3 over the industrial Cu/ZnO/Al_2O_3 catalyst at 210 °C and 60 bar. a, Recorded methanol mole fraction (black curves) during the injection of pulses with different NH_3 contents from 0 to 0.05%. **b**, Recorded methanol mole fraction (black curves) during the injection of pulses with different NH_3 contents from 0.075 to 0.5%.

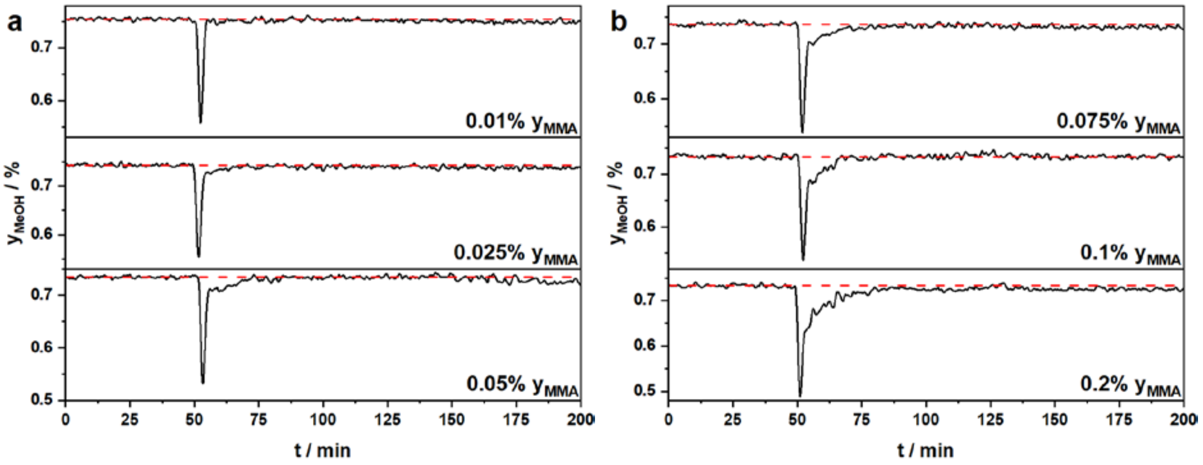

Supplementary Figure 2. HPPEs with monomethylamine (MMA) over the industrial Cu/ZnO/Al_2O_3 catalyst at 210 °C and 60 bar. a, Recorded methanol mole fraction (black curves) during the injection of pulses with different MMA contents from 0.01 to 0.05%. **b**, Recorded methanol mole fraction (black curves) during the injection of pulses with different MMA contents from 0.075 to 0.2%.

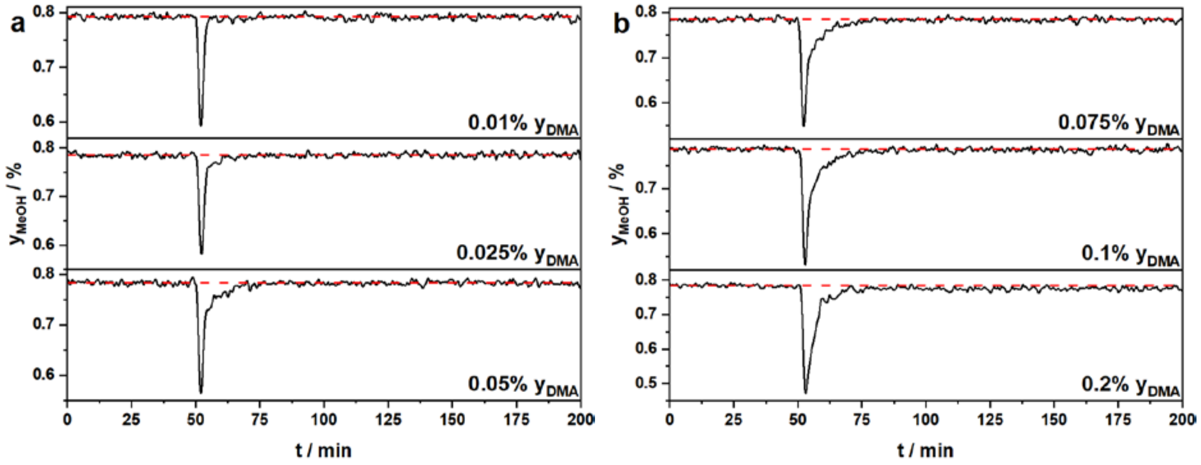

Supplementary Figure 3. 10 HPPEs with dimethylamine (DMA) over the industrial $Cu/ZnO/Al_2O_3$ catalyst at 210 °C and 60 bar. a, Recorded methanol mole fractions (black curves) during the injection of pulses with different DMA contents from 0.01 to 0.05%. **b**, Recorded methanol mole fractions (black curves) during the injection of pulses with different DMA contents from 0.075 to 0.2%.

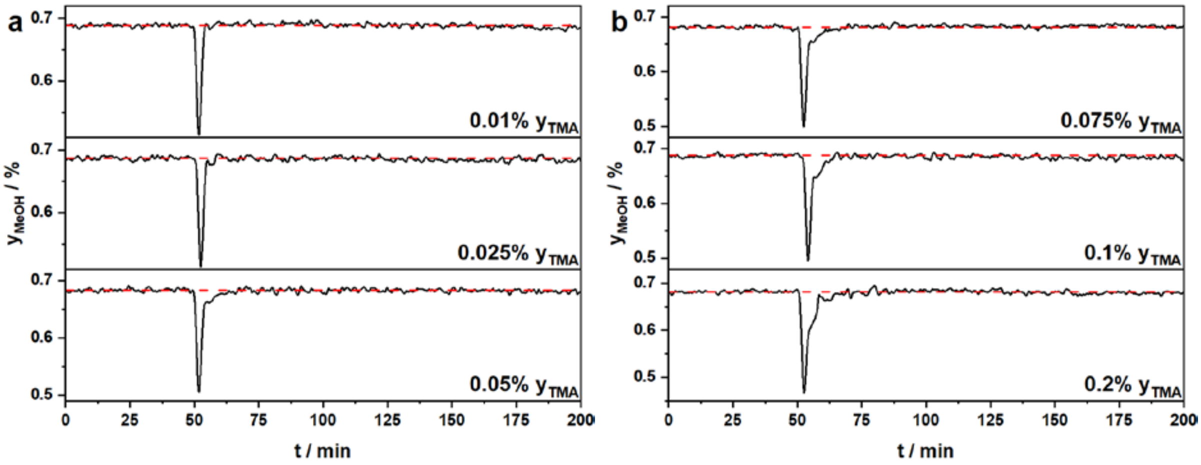

Supplementary Figure 4. HPPEs with trimethylamine (TMA) over the industrial $Cu/ZnO/Al_2O_3$ catalyst at 210 °C and 60 bar. a, Recorded methanol mole fraction (black curves) during the injection of pulses with different TMA contents from 0.01 to 0.05%. **b**, Recorded methanol mole fraction (black curves) during the injection of pulses with different TMA contents from 0.075 to 0.2%.

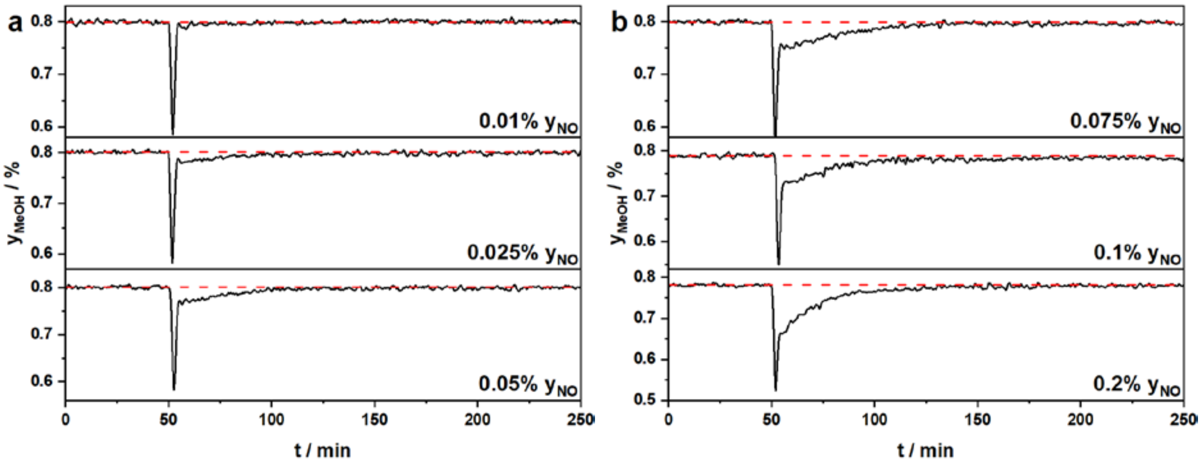

Supplementary Figure 5. HPPEs with NO over the industrial $Cu/ZnO/Al_2O_3$ catalyst at 210 °C and 60 bar. a, Recorded methanol mole fraction (black curves) during the injection of pulses with different NO contents from 0.01 to 0.05%. **b**, Recorded methanol mole fractions (black curves) during the injection of pulses with different NO contents from 0.075 to 0.2%.

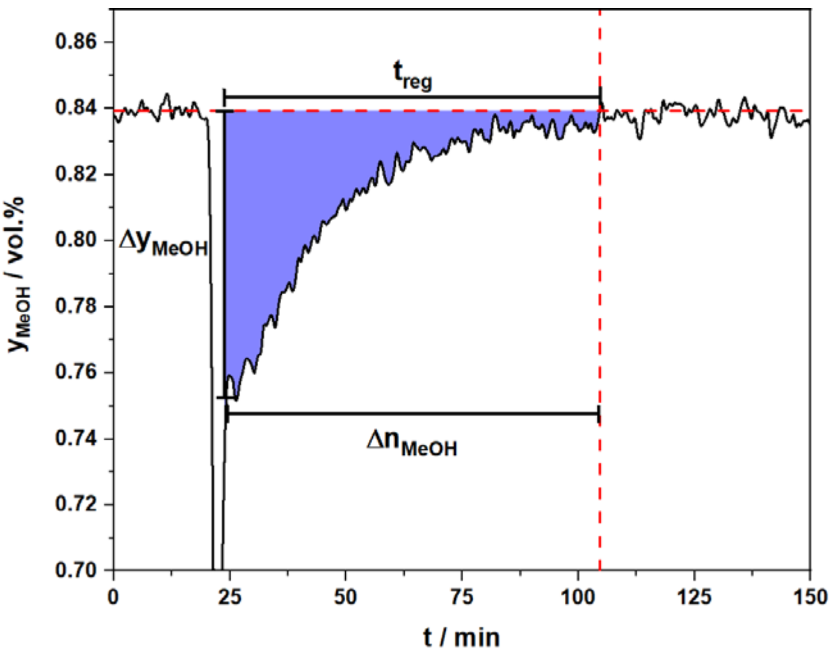

Supplementary Figure 6. Determination of the non-produced amount of methanol over a defined period of time (Δn_{MeOH}). The minimum methanol mole fraction after the temporary dilution (Δy_{MeOH}), the regeneration time until the initial methanol mole fraction is obtained again (t_{reg}) and the non-produced amount of methanol over a defined period of time (Δn_{MeOH}, blue area) for the HPPE with 0.1 ppm NH_3 from Supplementary Figure 1.b. The black curve describes the recorded methanol mole fraction in the product gas stream.

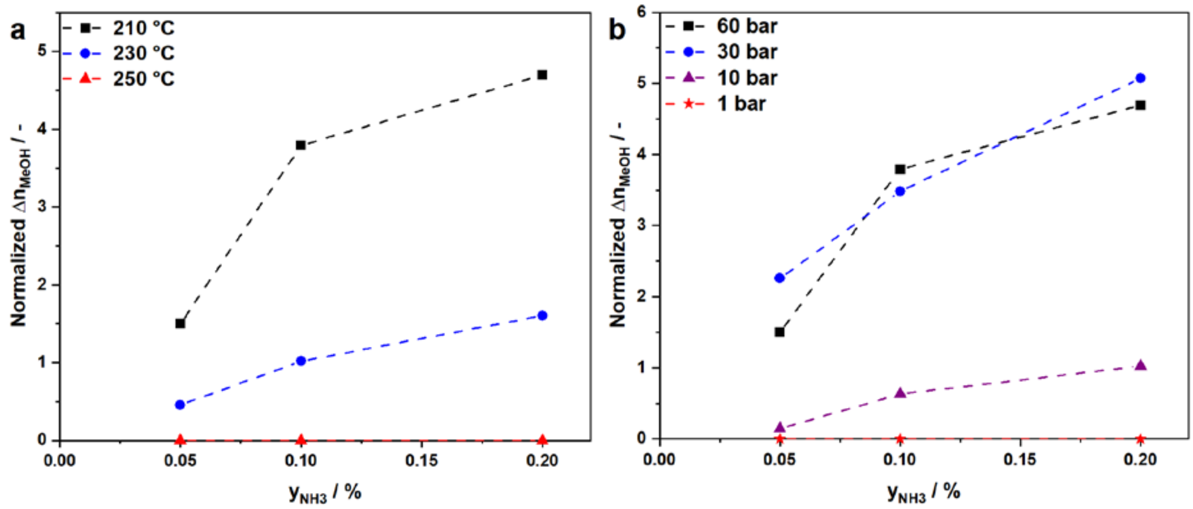

Supplementary Figure 7. Normalized Δn_{MeOH} values of HPPEs with NH_3 determined at different reaction conditions. a, Correlation of the normalized Δn_{MeOH} values against the injected mole fractions of NH_3 at constant pressure level (60 bar) and different reaction temperatures at 210 °C (black squares and line), 230 °C (blue points and line) and 250 °C (red triangles and line). The corresponding pulse experiments are shown in Supplementary Figure 8. **b**, Correlation of the normalized Δn_{MeOH} values with the injected mole fractions of NH_3 at constant reaction temperature (210 °C) and different pressures at 1 bar (red stars and line), 10 bar (violet triangles and line), 30 bar (blue points and line), 60 bar (black squares and line). The corresponding pulse experiments are shown in Supplementary Figure 9.

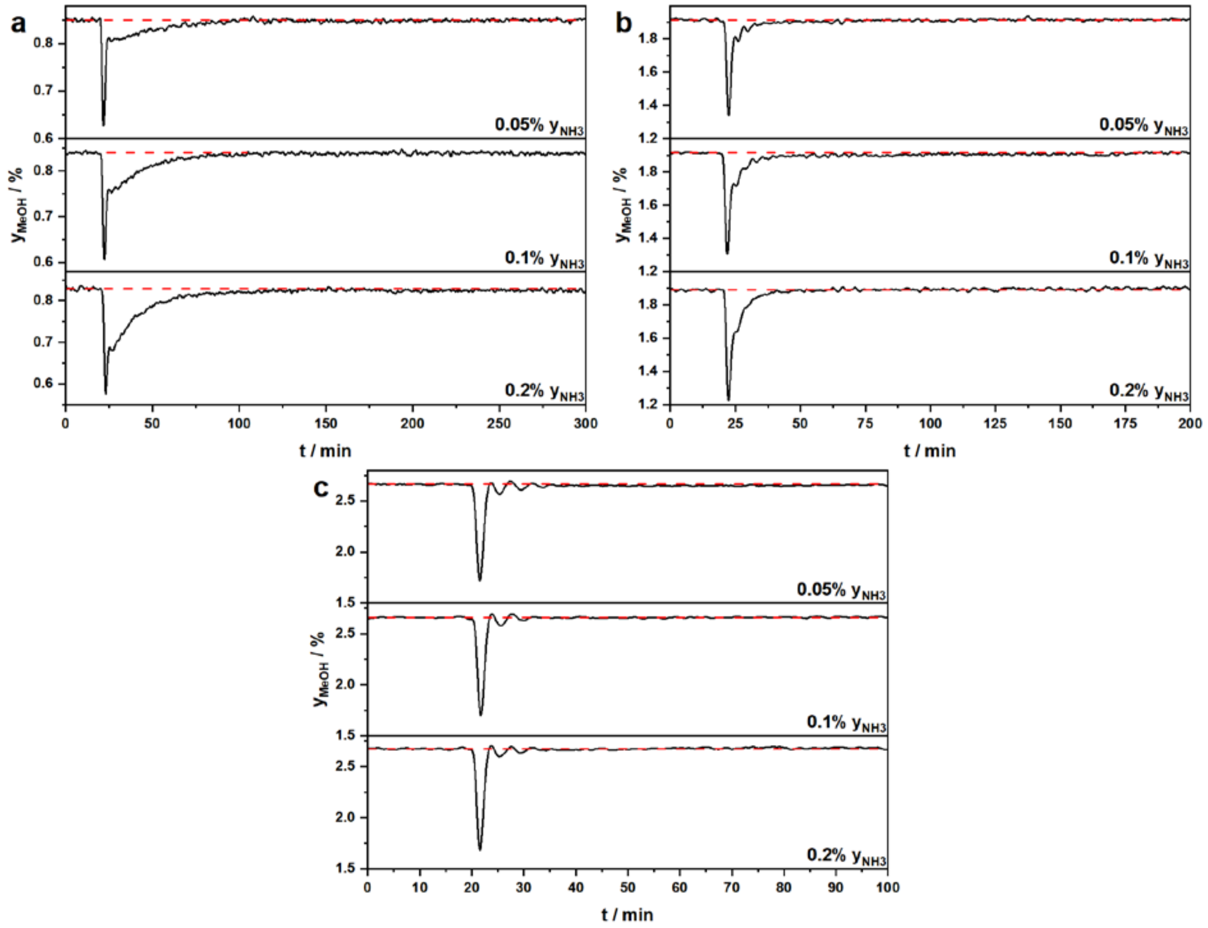

Supplementary Figure 8. HPPEs with NH_3 over the industrial Cu/ZnO/Al_2O_3 catalyst at constant pressure of 60 bar and different reaction temperatures. Recorded methanol mole fractions (black curves) during the injection of pulses with different NH_3 contents from 0.05 to 0.2% at **a** 210 °C, **b** 230 °C and **c** 250 °C.

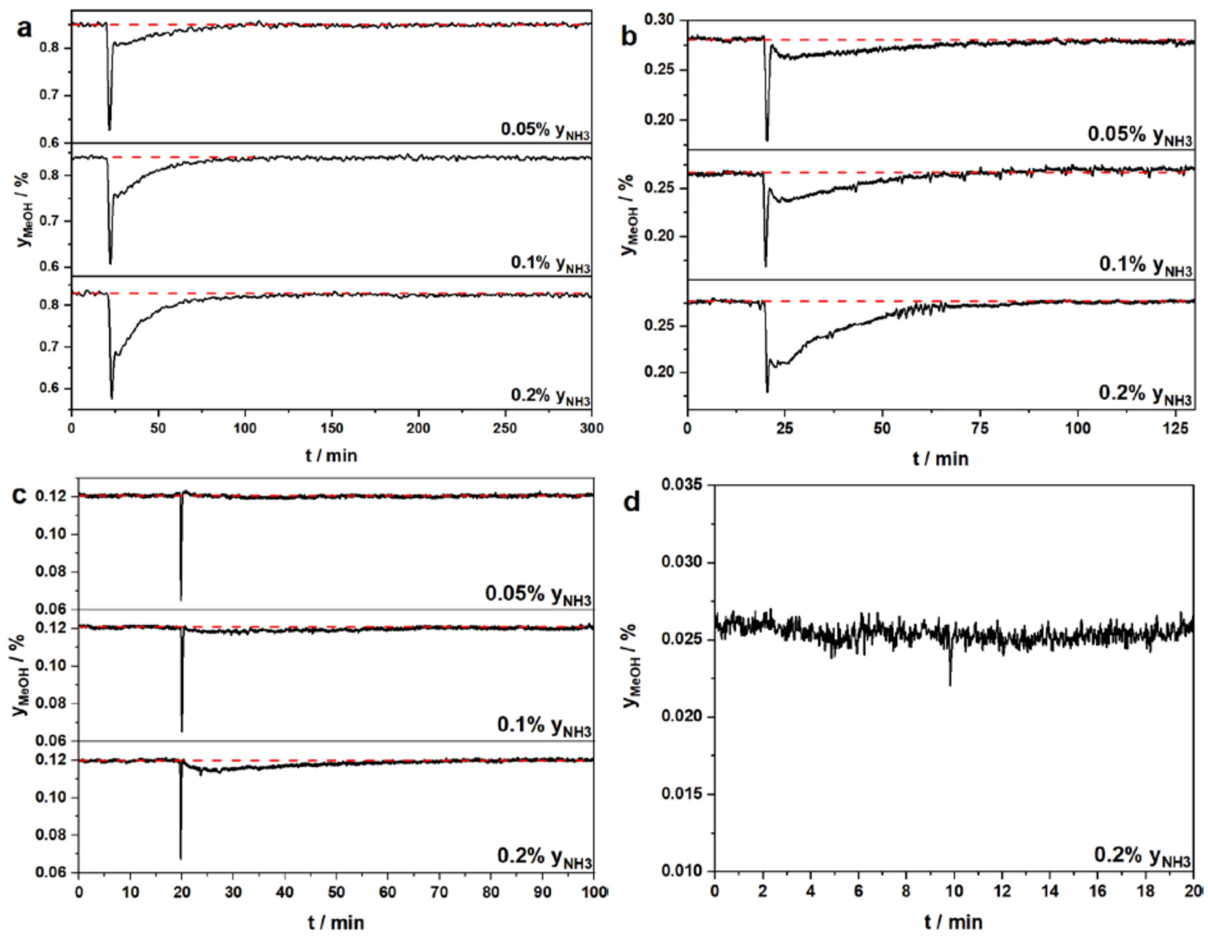

Supplementary Figure 9. HPPEs with NH_3 over the industrial $Cu/ZnO/Al_2O_3$ catalyst at constant reaction temperature of 210 °C and different pressures. Recorded methanol mole fractions (black curves) during the injection of pulses with different NH_3 contents from 0.05 to 0.2% at **a** 60 bar, **b** 30 bar, **c** 10 bar and **d** 1 bar.

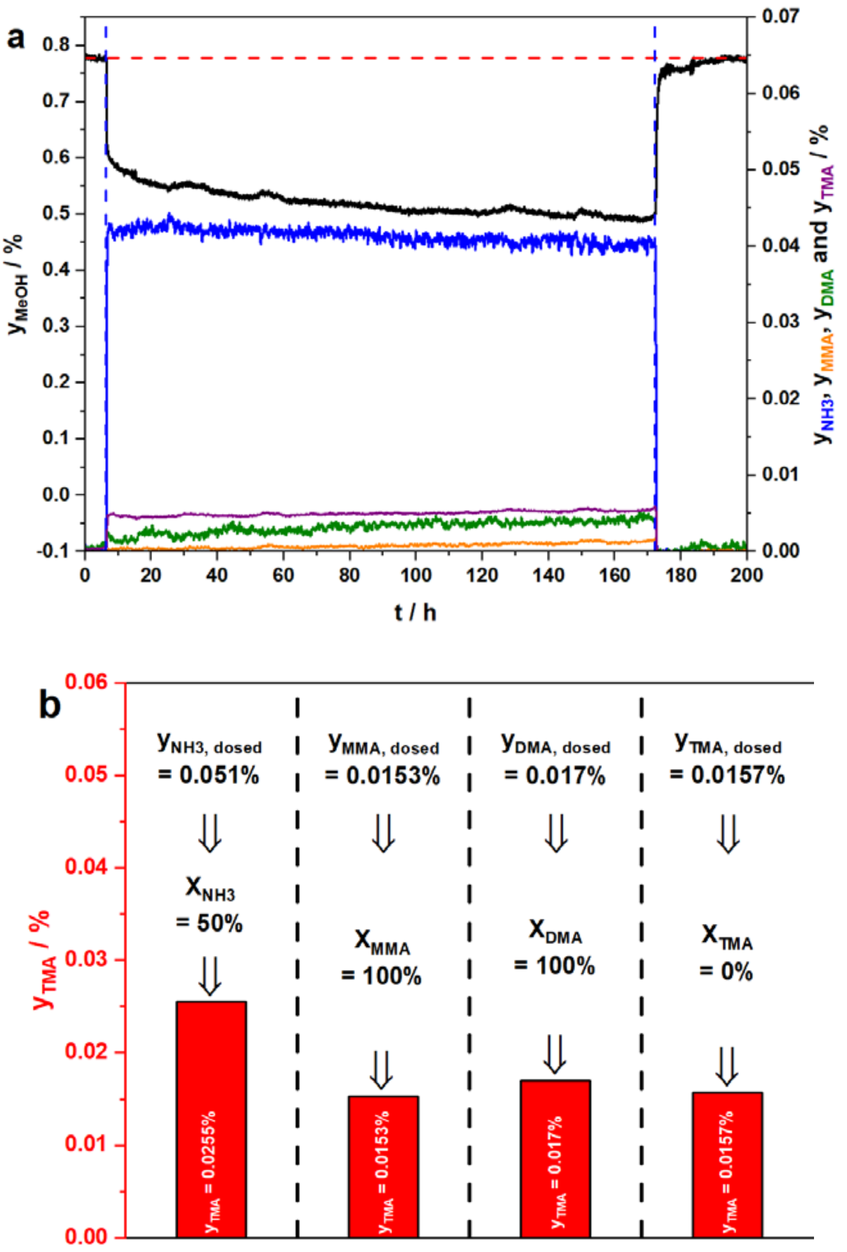

Supplementary Figure 10. Continuous dosing experiments with NH_3, MMA, DMA and TMA over the industrial $Cu/ZnO/Al_2O_3$ catalyst under different reaction temperatures. a, Recorded methanol (black curve), NH_3 (violet curve), MMA (orange curve), DMA (green curve) and TMA (blue curve) mole fractions during the continuous dosing of 0.05% NH_3 at 210 °C and 60 bar. **b**, Continuous dosing from left to right of 0.051% NH_3, 0.0153% MMA, 0.0170% DMA and 0.0157% TMA at 250 °C and 60 bar. The red bars describe the corresponding conversion degree to TMA.

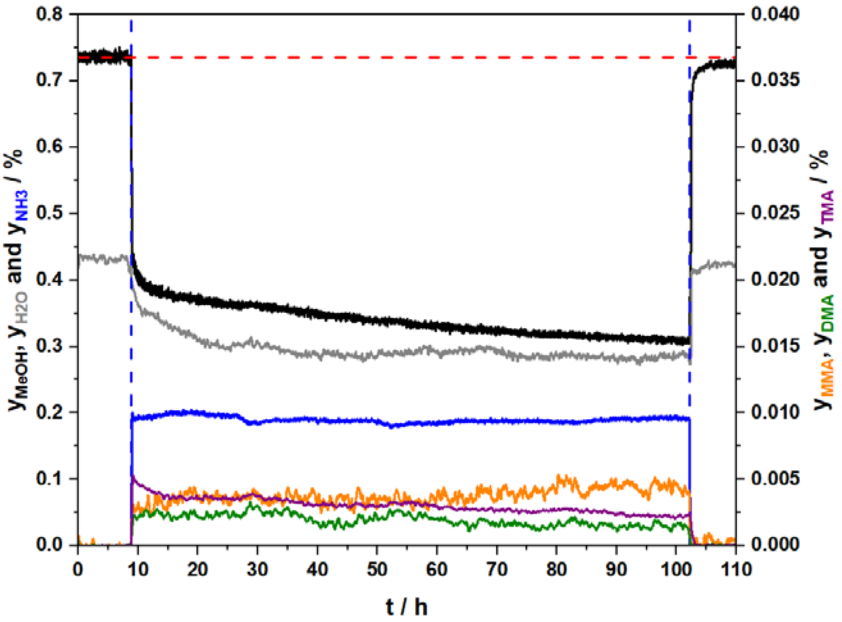

Supplementary Figure 11. Hydrogenation of NO to NH_3 and H_2O and further methylation of NH_3 to MMA, DMA and TMA over the industrial $Cu/ZnO/Al_2O_3$ catalyst at 210 °C and 60 bar. Recorded methanol (black curve), H_2O (grey curve), NH_3 (blue curve), MMA (orange curve), DMA (green curve) and TMA (violet curve) mole fractions.

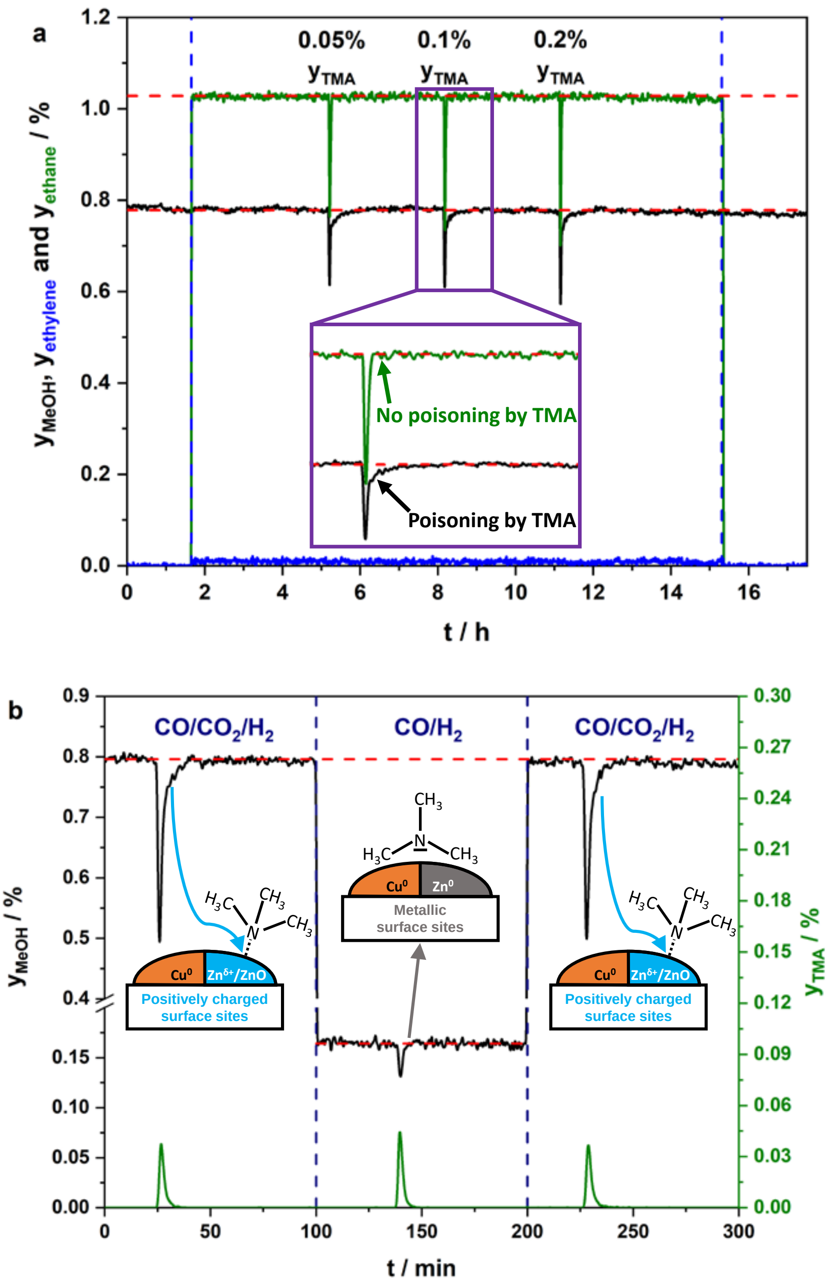

Supplementary Figure 12. Continuous dosing experiments and HPPEs for the identification of the surface sites of the industrial Cu/ZnO/Al$_2$O$_3$ catalyst. a, Continuous dosing of 1% of ethylene (blue curve), which is hydrogenated to ethane (green curve) under methanol (black curve) synthesis conditions, coupled with HPPEs with TMA. **b**, Syngas switching experiments from CO/CO$_2$/H$_2$ to CO/H$_2$ and back to CO/CO$_2$/H$_2$ over at 210 °C and 60 bar coupled with HPPEs with TMA. Here, the resulting methanol (black curve) and TMA (green curve) mole fractions in the product gas stream are shown. The images visualize the oxidative effect of CO$_2$ in the syngas mixture and the corresponding interaction of TMA with the catalyst surface. Orange marked area = Cu^0 sites, blue marked area = Zn$^{\delta+}$/ZnO sites, grey marked area = Zn^0 sites.

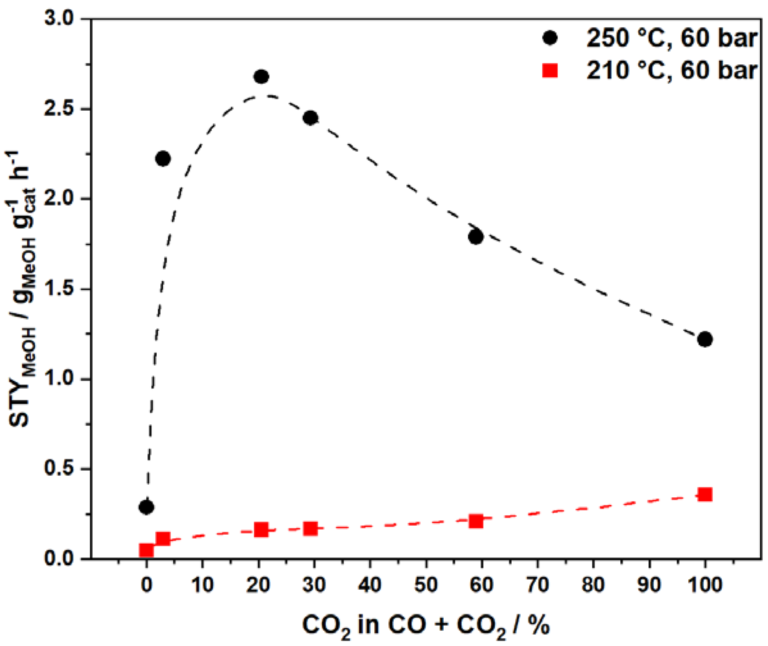

Supplementary Figure 13. Comparison of methanol productivities as a function of the CO_2 content in the syngas at different reaction temperatures. The black points and curve describe the methanol productivity at 250 °C and 60 bar and the red squares and curve at 210 °C and 60 bar.

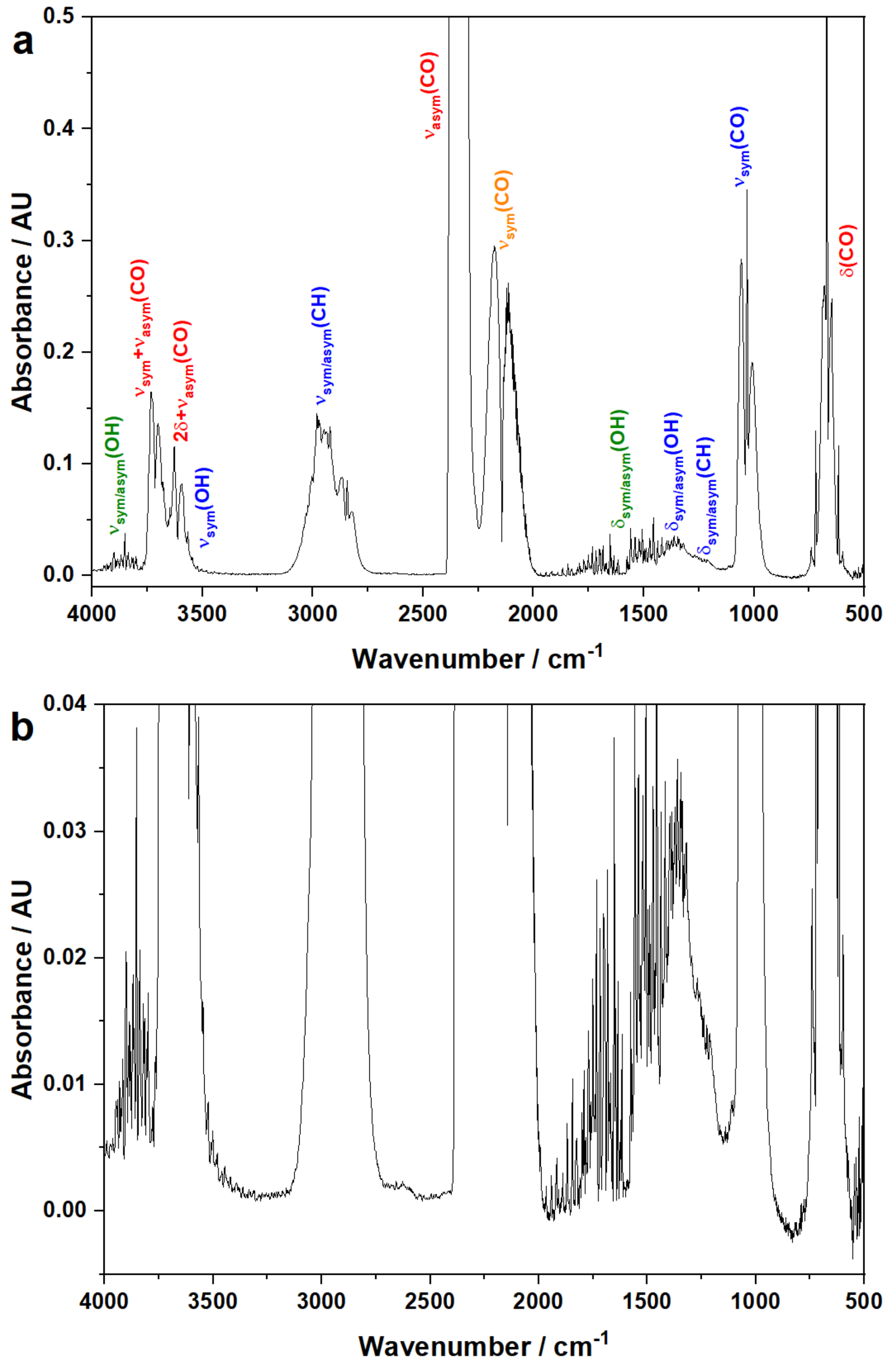

Supplementary Figure 14. IR spectrum (black curve) of the product gas stream after methanol synthesis over the industrial Cu/ZnO/Al_2O_3 catalyst at 210 C and 60 bar with the standard syngas mixture. a, All blue coloured vibration modes are from methanol, all red coloured from CO_2, all green coloured from water and the orange coloured mode from CO. ν = stretching vibration, δ = deformation vibration, δ + ν / ν + ν = combination vibrations, sym = symmetric, asym = asymmetric. **b**, Enlarged baseline region of the IR spectrum shown in **a**.

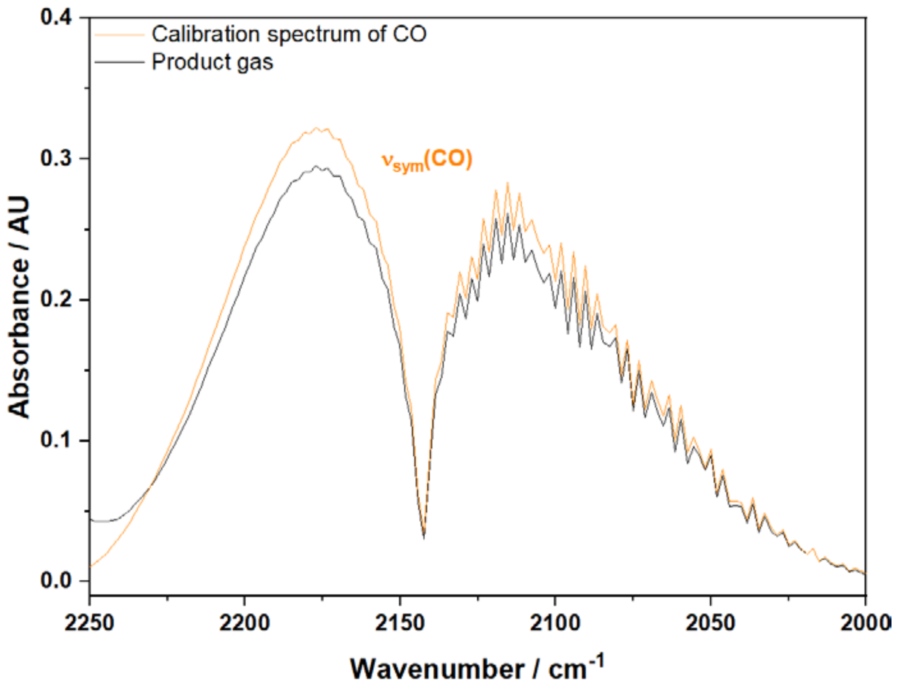

Supplementary Figure 15. IR spectrum of the product gas stream after methanol synthesis over the industrial $Cu/ZnO/Al_2O_3$ catalyst at 210 C and 60 bar with the standard syngas mixture. Here, the wavenumber region of the ν_{sym}(CO) vibration mode from the CO molecule is shown and the corresponding part of the product gas spectrum (black curve) is compared with a calibration spectrum of 13.5% CO (orange curve).

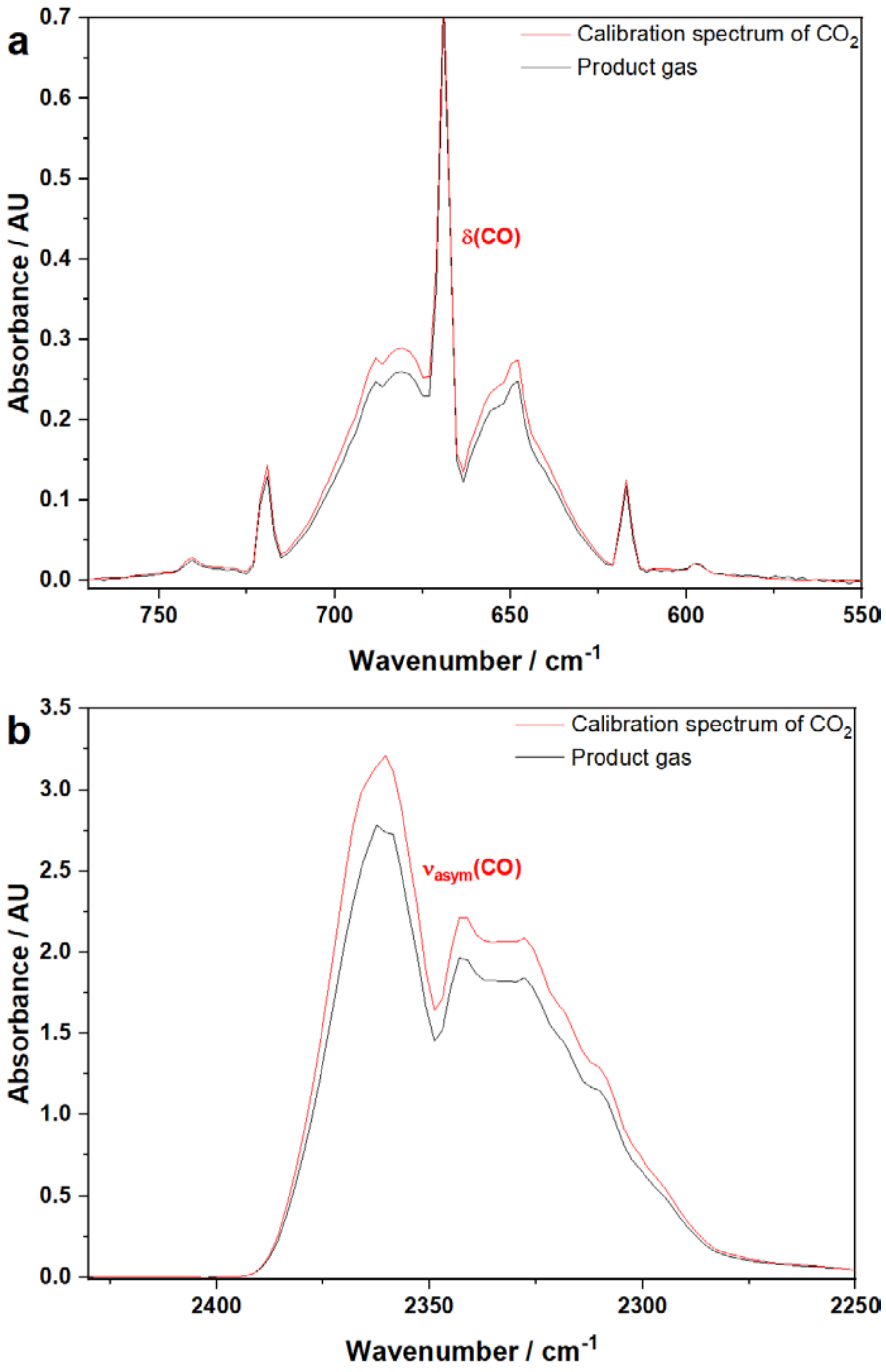

Supplementary Figure 16. IR spectrum of the product gas stream after methanol synthesis over the industrial Cu/ZnO/Al_2O_3 catalyst at 210 C and 60 bar with the standard syngas mixture. a, Wavenumber region of the δ(CO) vibration mode from the CO_2 molecule. **b,** Wavenumber region of the ν_{asym}(CO) vibration mode from the CO_2 molecule. The corresponding part of the product gas spectrum (black curves) is compared with a calibration spectrum of 3.5% CO_2 (red curves).

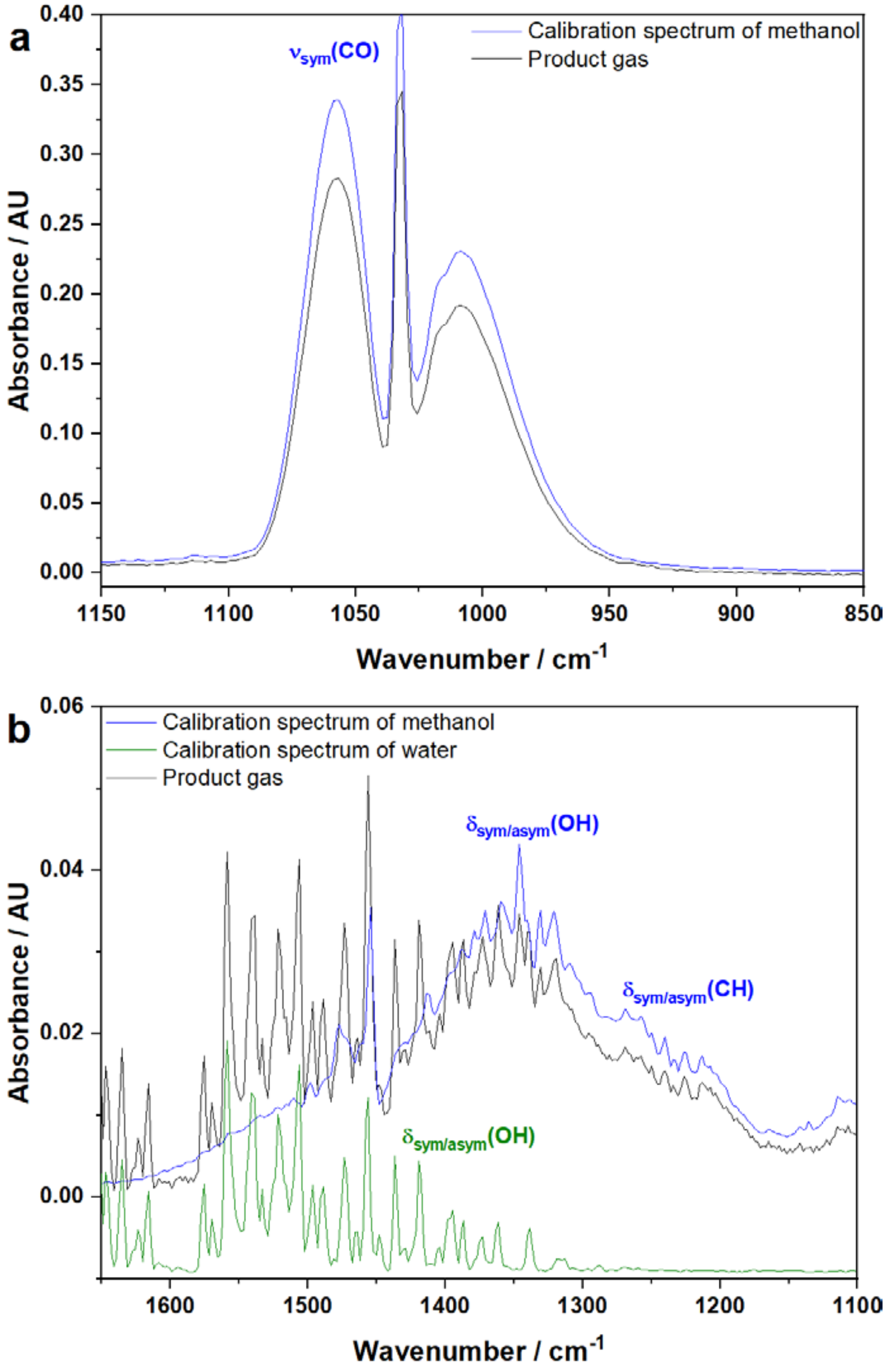

a
Calibration spectrum of methanol
Product gas
ν_{sym}(CO)
Absorbance / AU
Wavenumber / cm^{-1}
b
Calibration spectrum of methanol
Calibration spectrum of water
Product gas
$\delta_{sym/asym}$(OH)
$\delta_{sym/asym}$(CH)
$\delta_{sym/asym}$(OH)
Absorbance / AU
Wavenumber / cm^{-1}

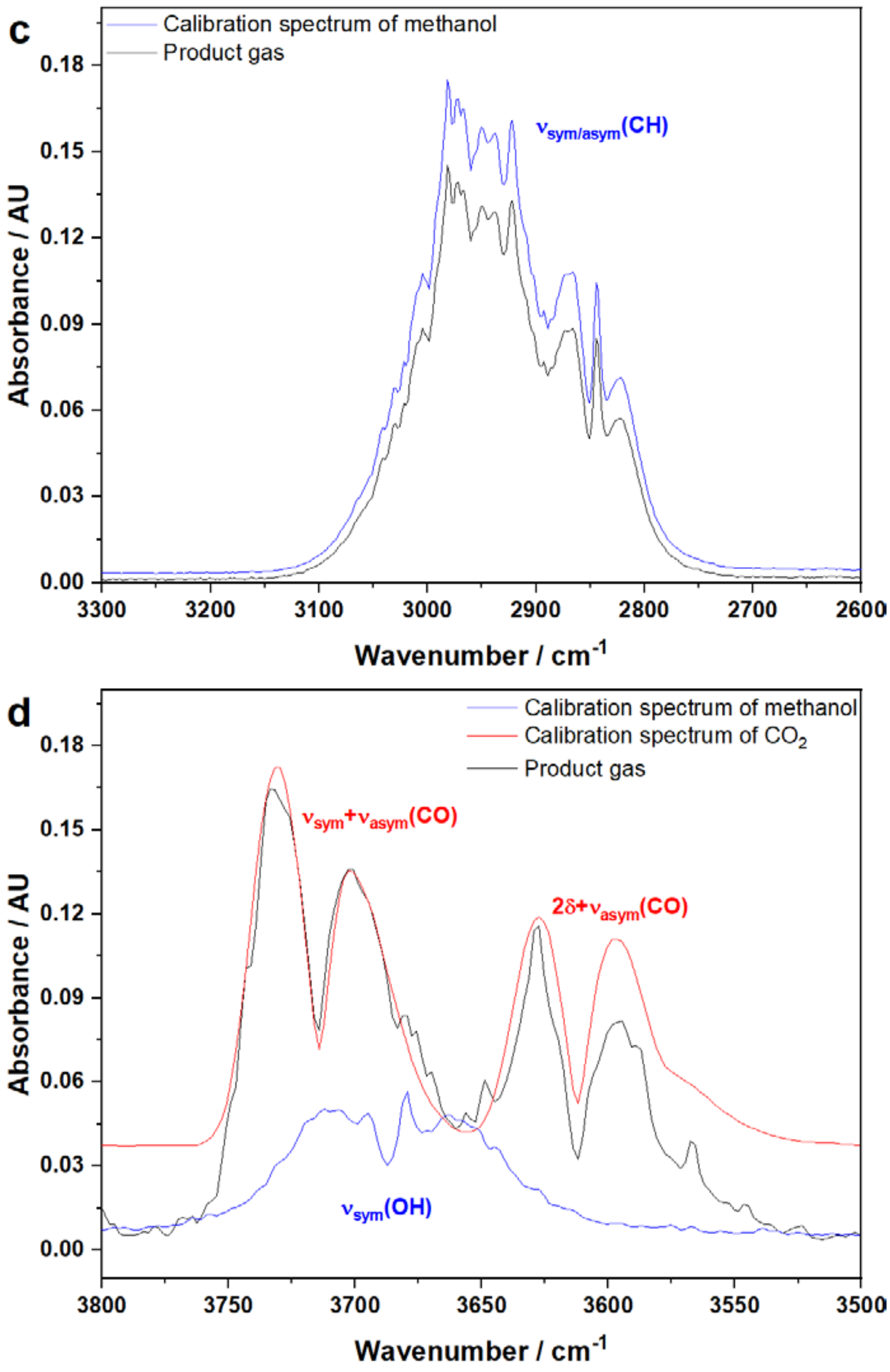

Supplementary Figure 17. IR spectrum of the product gas stream after methanol synthesis over the industrial Cu/ZnO/Al$_2$O$_3$ catalyst at 210 C and 60 bar with the standard syngas mixture. a, Wavenumber region of the ν_{sym}(CO) vibration mode from the methanol molecule. **b**, Wavenumber region of the $\delta_{sym/asym}$(OH) and $\delta_{sym/asym}$(CH) vibration modes from the methanol molecule. **c**, Wavenumber region of the $\nu_{sym/asym}$(CH) vibration mode from the methanol molecule. **d**, Wavenumber region of the ν_{sym} + ν_{asym}(CO), 2δ + ν_{asym}(CO) and ν_{sym}(OH) vibration modes from the methanol molecule. The corresponding part of the product gas spectrum (black curves) is compared with a calibration spectra of 1.0% methanol (blue curves), 0.4% water (green curve) and 3.5% CO_2 (red curve).

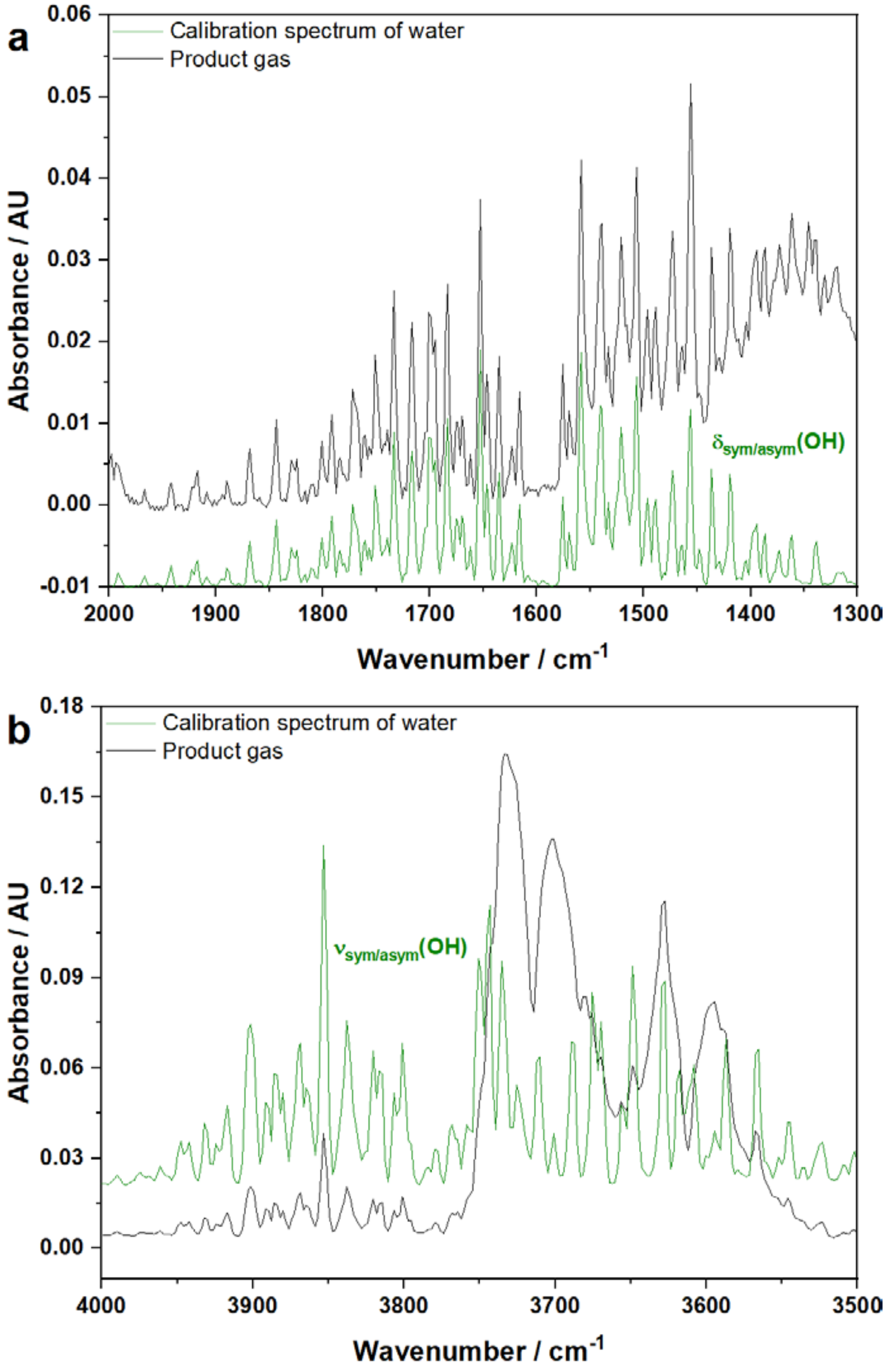

Supplementary Figure 18. IR spectrum of the product gas stream after methanol synthesis over the industrial Cu/ZnO/Al_2O_3 catalyst at 210 C and 60 bar with the standard syngas mixture. a, Wavenumber region of the $\delta_{sym/asym}$(OH) vibration mode from the H_2O molecule. **b**, Wavenumber region of the $\nu_{sym/asym}$(OH) vibration mode from the H_2O molecule. The corresponding part of the product gas spectrum (black curves) is compared with a calibration spectrum of 0.4% water (green curves).

Supplementary Notes

Supplementary Note 1. Analysis of the HPPEs: For the comparison of the different poisoning strengths, two pieces of information can be obtained from the pulse experiments (Supplementary Figure 6): The height between the initial and minimum methanol mole fraction after the temporary dilution (Δy_{MeOH}) and the regeneration time until the initial methanol concentration is obtained again (t_{reg}). We combined both pieces of information to obtain the non-produced amount of methanol over a defined period of time (Δn_{MeOH}) by integration. The limits for the integrated area are directly after the temporary dilution and before the initial methanol concentration is reached again.

Supplementary Note 2. Determination of the ideal reaction conditions for HPPEs: Supplementary Figure 7 shows the influence of reaction temperature and pressure on the observed strength of the reversible poisoning effect by NH_3. Here, pulse experiments were performed with different NH_3 mole fractions between 210 - 250 °C at constant 60 bar (Supplementary Figure 7a) and between 1 - 60 bar at constant 210 °C (Supplementary Figure 7b). The variation of the reaction parameters results in different production rates of methanol, so that the produced amount of methanol of all measurements was normalized to 1 for a better comparison of the resulting inhibition strengths. The normalized Δn_{MeOH} values were determined by integration as shown in Supplementary Figure 6 and correlated with the injected NH_3 concentrations. In both cases, temperature and pressure have a strong influence on the observed strength of the reversible poisoning. Decreasing temperatures and increasing pressure levels enhance the inhibition of methanol synthesis. As shown in Supplementary Figure 8, the reaction rate of the methylation and the conversion degree of NH_3 can be drastically increased by rising the temperature, so that the inhibition strength should be higher due to the higher consumption of formate intermediates. Besides, the desorption of the methylamines is preferred at higher temperatures and, consequently, the whole poisoning mechanism of NH_3 is significantly accelerated. In the case of HPPEs, the accelerated consumption of NH_3 and desorption of the methylamines lead to a shift of the minimum methanol mole fraction (Δy_{MeOH}) in the temporary dilution and to the reduction of the regeneration time (t_{reg}) (Supplementary Figure 8), which decreases the integrated area until it disappears at 250 °C. Thus, the acceleration of the reversible poisoning results in a lower inhibition effect on methanol formation. For the pressure variation (Supplementary Figure 7b), higher pressures favour the adsorption and re-adsorption probability of the reversible poison in the injected pulse. However,

HPPEs at 30 and 60 bar nearly reach the same normalized Δn_{MeOH} values. The reason for this could be that the saturation limit of NH_3 on the Cu/ZnO/Al_2O_3 catalyst is achieved at 30 bar and the further increase of the pressure results in the same ratio of the coverage degrees between NH_3 and syngas species. Another point is that the inhibition strength of NH_3 is equal to 0 at 1 bar as for the 250 °C measurement in Supplementary Figure 7a. At 1 bar, the methanol production rate is low as well as the concentration of the formate adsorbates and, therefore, also the tendency of NH_3 to adsorb on the surface. The resulting poisoning must be weak and an analysis with our analytics was not possible (Supplementary Figure 9d). As a result, the reaction temperature of 210 °C and the reaction pressure of 60 bar are ideal to investigate significantly strong reversible poisoning under industrially relevant methanol synthesis conditions. Consequently, all HPPEs in this work were performed under these conditions.

Supplementary Note 3. Analysis of by-product formation:

Supplementary Figure 14a shows the IR spectrum recorded with the online FTIR of the product gas stream after methanol synthesis over the industrial Cu/ZnO/Al_2O_3 catalyst at standard reaction conditions (210 °C, 60 bar, differential kinetic region, syngas: 13.5% CO, 3.5% CO_2, 73.5% H_2 and 9.5% N_2). The most relevant and intense bands of every IR-active compound (CO, CO_2, methanol, water) in the product gas stream are labelled using the corresponding vibration mode. Supplementary Figure 14b shows the baseline of the IR spectrum (Supplementary Figure 14a) in detail. In addition, Supplementary Figures 15 to 18 show every vibration mode of the main compounds in detail, and these bands are compared with the calibration spectra of the corresponding compound. For the recording of the calibration spectra, a set of calibration standards was prepared including one of the gaseous compounds CO, CO_2, methanol or water in N_2. These standards were passed through the gas cell of the FTIR and the corresponding calibration spectrum was recorded. The analysis of the mentioned figures leads to the conclusions that the product gas stream of methanol synthesis under standard conditions only contains the four IR-active compounds CO, CO2, methanol and water and that the presence of significant amounts of by-products (formaldehyde, ethanol, dimethyl ether) can be excluded.
