# Supplementary material for: Identifying the nature of the active sites in methanol synthesis over Cu/ZnO/Al2O3 catalysts
Source: Nat Commun. 2020 Aug 4;11:3898. doi: 10.1038/s41467-020-17631-5 (PMC7403733; doi:10.1038/s41467-020-17631-5)
Supplement: Supplementary file 2 — Peer Review File [file 41467_2020_17631_MOESM2_ESM.pdf]

Editorial Note: This manuscript has been previously reviewed at another journal that is not operating a transparent peer review scheme. The manuscript was considered suitable for publication without further review at *Nature Communications.*
